# Function Space Optimization: A Symbolic Regression Method for Estimating Parameter Transfer Functions for Hydrological Models

**DOI:** 10.1029/2020WR027385

**Published:** 2020-10-06

**Authors:** M. Feigl, M. Herrnegger, D. Klotz, K. Schulz

**Affiliations:** ^1^ Institute for Hydrology and Water Management University of Natural Resources and Life Sciences Vienna Austria; ^2^ LIT AI Lab and Institute for Machine Learning Johannes Kepler University Linz Linz Austria

**Keywords:** regionalization, machine learning, rainfall‐runoff modeling, transfer functions, optimization, distributed models

## Abstract

Estimating parameters for distributed hydrological models is a challenging and long studied task. Parameter transfer functions, which define model parameters as functions of geophysical properties of a catchment, might improve the calibration procedure, increase process realism, and can enable prediction in ungauged areas. We present the function space optimization (FSO), a symbolic regression method for estimating parameter transfer functions for distributed hydrological models. FSO is based on the idea of transferring the search for mathematical expressions into a continuous vector space that can be used for optimization. This is accomplished by using a text generating neural network with a variational autoencoder architecture that can learn to compress the information of mathematical functions. To evaluate the performance of FSO, we conducted a case study using a parsimonious hydrological model and synthetic discharge data. The case study consisted of two FSO applications: single‐criteria FSO, where only discharge was used for optimization, and multicriteria FSO, where additional spatiotemporal observations of model states were used for transfer function estimation. The results show that FSO is able to estimate transfer functions correctly or approximate them sufficiently. We observed a reduced fit of the parameter density functions resulting from the inferred transfer functions for less sensitive model parameters. For those it was sufficient to estimate functions resulting in parameter distributions with approximately the same mean parameter values as the real transfer functions. The results of the multicriteria FSO showed that using multiple spatiotemporal observations for optimization increased the quality of estimation considerably.

## Key Points

- This study introduces a method to infer transfer functions for the multiscale parameter regionalization approach
- We demonstrate its ability in a case study using synthetic runoff data
- We show that multicriteria optimization can improve the estimation of parameter transfer functions

## 1 Introduction

Distributed hydrological models are widely used tools to model spatiotemporal processes in catchments. The modeled processes include the simulation of spatially distributed land surface fluxes (e.g., Rakovec et al., 2016), estimating the hydrological response to climate change (e.g., Hattermann et al., 2017; Kay et al., 2015) or hydrological response to land use changes (e.g., Hundecha & Bárdossy, 2004; Wijesekara et al., 2012). In general, process‐based distributed hydrological models can be classified in two groups: conceptual models and physically based models (Devia & Ganasri, 2015). Both depend to some extend on parameter calibration (Beven, 2001; Kirchner, 2006). Thus, in practice both approaches need to be calibrated and demand substantial expertise. Physically based models frequently lack observations necessary to define parameters correctly. In such situations, the uncertain parameters are either treated as physical constants (Clark et al., 2017), that is, a fixed value for a larger area, or are optimized as well. Both methods most likely result in reduced process realism, while still producing reasonable runoff predictions.

A solution to retain the process realism of hydrological models is to relate landscape properties to hydrologic behavior (Clark et al., 2016). This can be accomplished by using geophysical information for defining model parameters. This is however nontrivial. As a matter of fact, Clark et al. (2017) described this as one of the major unsolved challenges in hydrologic parameter estimation. Most recently Blöschl et al. (2019) also mention the “disentanglement and reduction of structural/parameter/input uncertainty in hydrological models” as one of the 23 unsolved problems in hydrology. Defining model parameters using the spatially distributed geophysical properties of a basin would reduce parameter uncertainty, increase process realism and the predictive ability of the model, and allow for runoff prediction in ungauged basins. This challenge is closely related to the idea of regionalization, which can be summarized as the geographical migration of hydrological model structures (Buytaert & Beven, 2009). Due to the problem of parameter equifinality in hydrological models (Beven, 2006), finding a relationship after parameter optimization might result in a weak or false regionalization (Hundecha & Bárdossy, 2004; Kumar et al., 2013; Samaniego et al., 2010). To prevent this, the *simultaneous regionalization* method (Abdulla & Lettenmaier, 1997; Hundecha & Bárdossy, 2004; Parajka et al., 2005) was developed. This method tries to overcome this restriction by defining the relationship a priori in form of a *transfer function* and evaluating it in a set of validation basins.

Samaniego et al. (2010) introduced the multiscale parameter regionalization (MPR) as an extension of simultaneous regionalization. MPR defines the parameters on the scale of geophysical observations before aggregating them to the model scale, thus including small‐scale variations in their computed parameters. Instead of defining transfer functions using a regression approach, they define mathematical functions of geophysical properties of a catchment in MPR. This results in a constrained form of parameter calibration that preserves the physical interpretation of the parameter values and produces seamless parameter fields, that is, they do not exhibit artificial spatial discontinuities often observed in distributed hydrological models (Samaniego et al., 2017).

These mathematical functions are usually unknown (in many cases we do not even know if they exist in the first place). Hence, the main restriction of MPR today is the selection of suitable parameter transfer functions (Samaniego et al., 2017). Potential candidates for transfer functions for hydrological models could be pedotransfer functions. They relate soil properties to soil parameters and were already investigated extensively in the past (see, e.g., Van Looy et al., 2017). Besides those, functional relationships between model parameters and geophysical properties are not well known and we still lack methods that perform adequate estimation. At the same time, we assume that they exist.

Klotz et al. (2017) were the first to investigate a symbolic regression approach to automatically estimate transfer functions. The term symbolic regression refers to methods that search the space of mathematical expressions while minimizing some error metrics, usually based on evolutionary computation (Bongard & Lipson, 2007; Cornforth & Lipson, 2015; Schmidt & Lipson, 2009). By using a simple model and synthetic data, Klotz et al. (2017) showed that it is possible to automatically estimate transfer functions from stream data in a virtual setting.

While the general idea of Klotz et al. seemed to work, it had two main difficulties: a bias toward overly simple transfer functions and the need to solve a difficult high dimensional discrete optimization problem. Both problems result from the representation of transfer functions as a discrete vector of a context free grammar (CFG). These limitations will be explained in detail in the methods part of this publication and are a main motivation for this work.

To overcome these limitations, the proposed method is based on the interpretation of mathematical functions as text, where each symbol of a function is seen as a “word.” Recent developments in Natural Language Processing (NLP) resulted in powerful Artificial Intelligence (AI) architectures which are able to translate (e.g., Srivastava et al., 2018), generate (e.g., Lu et al., 2018) and classify (e.g., Yang et al., 2019) text. While most symbolic regression methods are based on evolutionary algorithms, the method presented here is based on transferring the semantic information of text into a continuous space to adequately define nearness between functions. This is accomplished by a text generating neural network. The advantage we expect is that the search becomes more efficient and unbiased due to the continuous space and its properties. To our knowledge, only Gómez‐Bombarelli et al. (2018) investigated an approach with a similar idea where they transferred discrete representations of molecules into a continuous vector representation. The application we present is focused on a relevant problem in the hydrological sciences; nevertheless, it could potentially also be applied to other fields where functional relationships have to be derived from observational data.

The search for parameter transfer functions is a complex task; therefore, investigating ways to reduce its complexity and further constrain it is desirable. In recent years, multiple publications showed the value of using observations of spatially distributed fluxes and storage components for parameter calibration, additionally to stream data (e.g., Baroni et al., 2019; Demirel et al., 2018; Francke et al., 2018; Huang et al., 2019; Nijzink et al., 2018; Rakovec et al., 2016; Stisen et al., 2011, 2018; Zink et al., 2018). Those additional observations can be included in the optimization procedure by a multicriteria objective function. This constrains the parameter optimization and can improve the representation of hydrologic states and fluxes in a model (Zink et al., 2018). In this publication we will investigate the usefulness of multicriteria optimization for finding transfer functions.

This publication presents the function space optimization (FSO) as a method for estimating parameter transfer functions for distributed hydrological models and applies the FSO method in a case study. The case study uses synthetic runoff data and consists of three tests. In the first test only runoff data are used for estimating transfer functions. In the second and third tests we investigate how the use of additional spatial‐temporal information in a multicriteria optimization can improve the transfer function estimation.

## 2 Methods

### 2.1 The MPR Method

Let the two spatial scales used in the MPR approach be denoted by O and M, for the spatial scales of observations and the model, respectively. Note that O<M is a necessary condition for MPR. Let $\theta^{O}\in {\mathbb{R}}^{n}$ be the model parameters on the spatial scale of observation, defined by

(1) $$\theta^{O}=f_{\mathrm{tf}}\left(X^{O},\beta \right).$$

We call *f*_*tf*_ : *ℝ*^*n* × *sp*^ → *ℝ*^*n*^ a *transfer function*. It uses a set of k numerical parameters *β* ∈ *ℝ*^*k*^ to map the matrix $X^{O}$ to $\theta^{O}$. Here, *n* ∈ *ℕ* is the number of grid cells defining the catchment. The matrix $X^{O}\in {\mathbb{R}}^{n\times \mathrm{sp}}$ contains the *sp* ∈ *ℕ* physical properties of the catchment on the spatial scale of observations for each grid cell. We refer to those properties as *spatial predictors*. While $\theta^{O}$ and $X^{O}$ are spatially distributed, *β* is a vector of global parameters.

Model parameters on the spatial scale of the model, $\theta^{M}\in \mathbb{R}$, can thus be defined as

(2) $$\theta^{M}=f_{a}\left(\theta^{O}\right),$$

where *f*_*a*_ denotes an *aggregation* function which upscales the values of $\theta^{O}$ to $\theta^{M}$. Theoretically, any kind of aggregation function is possible. Samaniego et al. (2010) give the following examples for possible upscaling functions: arithmetic mean, geometric mean, harmonic mean, maximum difference, and the majority. There are no explicit averaging rules for various model parameters (Samaniego et al., 2010), and in the case of no existing theories, trying different basins and spatial scales might be the only procedure to identify them adequately (Samaniego et al., 2017). Another possible approach was described in a recent publication by Schweppe et al. (2019). They implemented MPR using the generalized mean with the form $M_{p}\left(x_{1},\ldots ,x_{n}\right)={\left(\frac{1}{n}\sum_{i=1}^{n}x_{i}^{p}\right)}^{\frac{1}{p}}$. The exponent *p* ∈ *ℝ* can be interpreted as a weighting, which gives either more importance to large values (*p* > 1) or smaller values (*p* < 1). The special case of *p* = 1 is the arithmetic mean. This general form of averaging can be optimized and therefore included in any optimization routine.

The problem of inferring the transfer of spatial predictors to model parameters can roughly be divided into two parts: (a) finding the correct transfer function and global parameters and (b) finding the correct aggregation function. We here apply the arithmetic mean aggregation and focus mainly on the estimation procedure of transfer functions and global parameters. For (b), one can either define an aggregation function using knowledge from previous investigations about the parameter, trying different aggregation functions, or use the generalized mean as an additional parameter to optimize. It is important to note that the estimated transfer functions are valid for the chosen aggregation function, and it cannot generally be assumed that they perform equally well with other types of aggregation.

With this we can define the aim of any transfer function estimation procedure:

(3) $$arg\min_{f_{\mathrm{tf}},\beta }\varepsilon =\arg\min_{f_{\mathrm{tf}},\beta }\;f_{\text{loss}}\left(Q_{\mathrm{sim}},Q_{\mathrm{obs}}\right),$$

where *ε* is the model loss, defined by a loss function *f*_*loss*_, which is dependent on the model simulated discharge *Q*_*sim*_ and the observed discharge *Q*_*obs*_. A suitable loss function must be chosen depending on the specific problem, for example, Nash‐Sutcliffe efficiency (Nash & Sutcliffe, 1970) for rainfall‐runoff modeling.

Considering these definitions, assumptions, and restrictions, we developed a method to infer parameter transfer functions and their global numerical parameters simultaneously from data.

### 2.2 FSO

The main difficulty of inferring *f*_*tf*_  lies in the transfer of the task into an optimizable problem. In general, this means transferring it into a searchable numerical space. To make it searchable, close points in this space should also be close in their loss function, hence producing a smooth response surface. Since it is not possible to estimate the loss functions of all relevant transfer functions (a case where optimization would not be necessary), we have to find other properties which induce this closeness of loss function.

This leads to the main idea of FSO: define a numerical space which defines distance between functions by (1) semantic closeness and (2) closeness in the resulting parameter distributions. Property (1) specifically includes the interpretation of functions as text, in which function symbols (e.g., “+”, “−“, and “elevation”) are interpreted as words. Property (2) is necessary since physical catchment properties are often highly correlated. Hence, the functions “*sand* × 0.3+1.3“and “*clay* ×  − 0.38+1.6“produce nearly the exact same parameters, even though their semantics are different.

Property (2) implies the a priori choice of global parameters *β* (the numerical values in the function) and results in distinguishing *f*_*tf*_ also by their specific *β* values. This allows for the simultaneous optimization of *f*_*tf*_ and *β*, since they are both represented in the numerical space. For brevity, we will use the term *f*_*tf*_ or transfer function, as a synonym for *f*_*tf*_ and *β*.

By transferring the problem in a numerical space with the above‐mentioned properties, any continuous global optimization method would be applicable. The steps for creating such a space and its use in the estimation of a transfer function will be described in the following sections.

#### 2.2.1 Defining Relevant Transfer Functions

**Figure 1:**
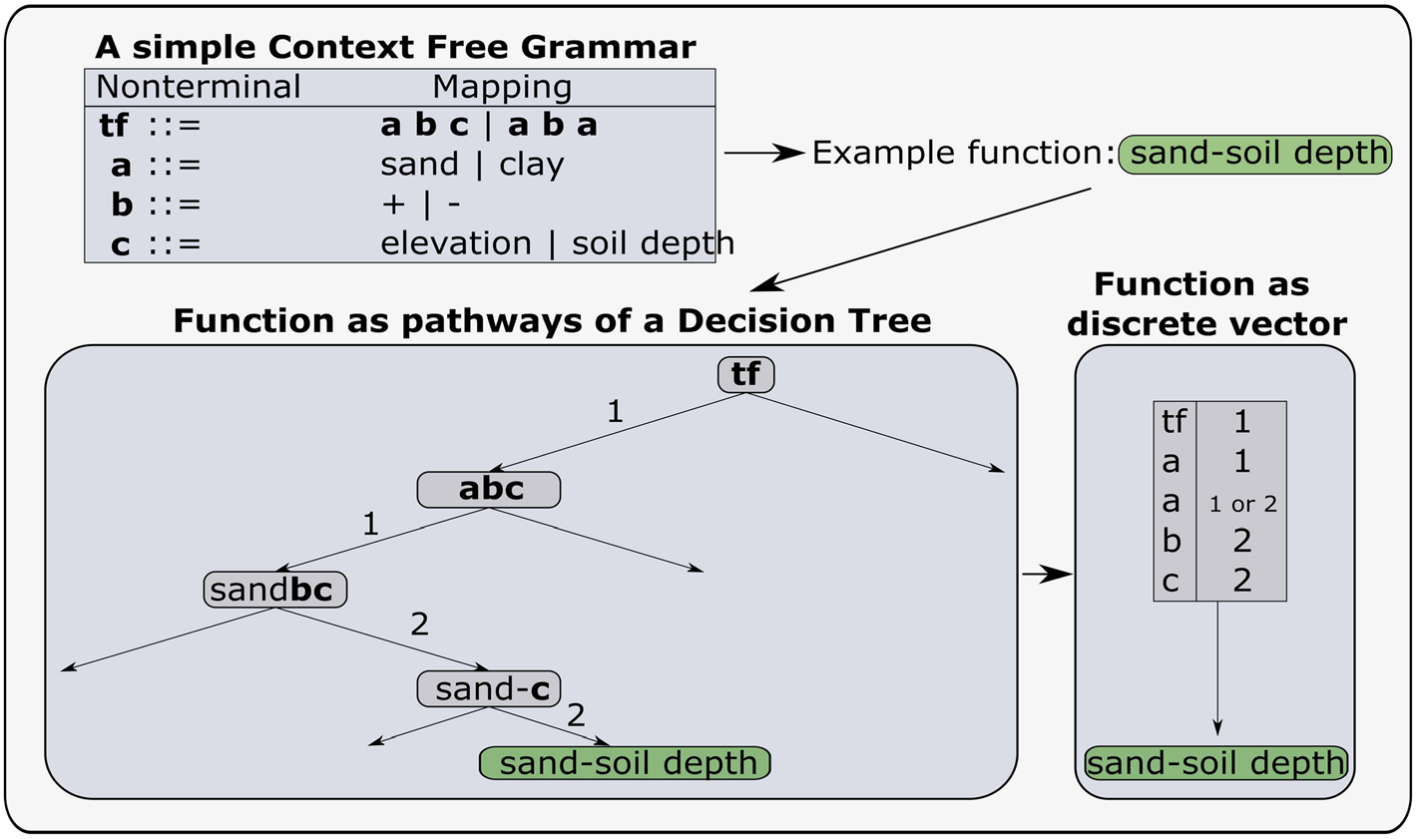
Example of a simple context free grammar (CFG) with an example function in its decision tree and vector representation.

In the first step of creating a search space, it is necessary to define a realm of possible transfer functions. A context‐free grammar (CFG) (Knuth, 1965) is used for this purpose. In general, a CFG is a set of variables, operators, and structural rules that can produce strings. It consists of nonterminal symbols and their corresponding mapping. A nonterminal symbol, in contrast to a terminal symbol, refers to a symbol that can still be further evaluated in the CFG (i.e., it has a mapping). An example of a simple CFG is given in Figure 1. A detailed and formal definition of CFGs can be found in Klotz et al. (2017).

In the simple example in Figure 1 only two options are available for all nonterminals. However, in any actual application this number will be larger. The nonterminals of a CFG can be interpreted as pathways in a decision tree, and the corresponding pathway options can be used to represent a function as a discrete vector. The lower left part of Figure 1 shows how a function can be derived from the CFG by choosing a certain pathway in its decision tree representation. The lower right part shows how the same function can be represented as a discrete vector. The entries of this vector correspond to the chosen option for each of the nonterminal symbols. The discrete vector has the same length for every function in a grammar. Therefore, if a nonterminal (e.g., the second **a** in the vector representation in Figure 1) is not used in a function pathway, its entry is not affecting the resulting function. For that reason, one of the entries of the discrete vector in Figure 1 can either be 1 or 2 and still produce the same function.

Klotz et al. (2017) used the vector representation of transfer functions as search space for solving the problem of *f*_*tf*_ estimation. Such process converts the search for the optimal transfer function into a discrete optimization problem. Even though this is a straightforward approach, it results in an ill‐defined optimization space and a bias toward very simple solutions. Both issues result from the properties of the vector representation of a CFG. For simplicity we will here refer to the CFG vector representation of a transfer function as *V*_*CFG*_.

Looking at the characteristics of *V*_*CFG*_ regarding its ability to map functions to integers, two important properties can be noticed: (1) any distance metric for numerical vectors (e.g., Euclidean distance) does not reflect the closeness of the resulting functions in the objective function and (2) the representation of a function as *V*_*CFG*_ is not unique. Both properties result from the fact that we use a discrete vector to represent a directed graph.

These two properties influence the optimization of *V*_*CFG*_ significantly. Property (1) results in a very difficult and ill‐posed optimization problem, considering that close points in the vector space most likely will not reflect similar results in terms of the objective function. Property (2) results in an optimization problem which is strongly biased toward simple functions. Simple functions can generally be represented with less dimensions than more complex ones. Since *V*_*CFG*_ has the same dimensionality for all functions, this results in a large part of the *V*_*CFG*_ that has no effect on the resulting functions. Hence, many different *V*_*CFG*_ will produce the same function. The resulting increased probability of finding simpler functions compared to more complex functions leads to a bias in the optimization.

These issues were one of the main motivations for developing FSO. The main advantage of using a CFG for FSO is the possibility of sampling functions, while preserving the defined function properties. Thereby, we use it to create a (very large) realm of possible function for the transfer function search.

#### 2.2.2 Variational Autoencoder

**Figure 2:**
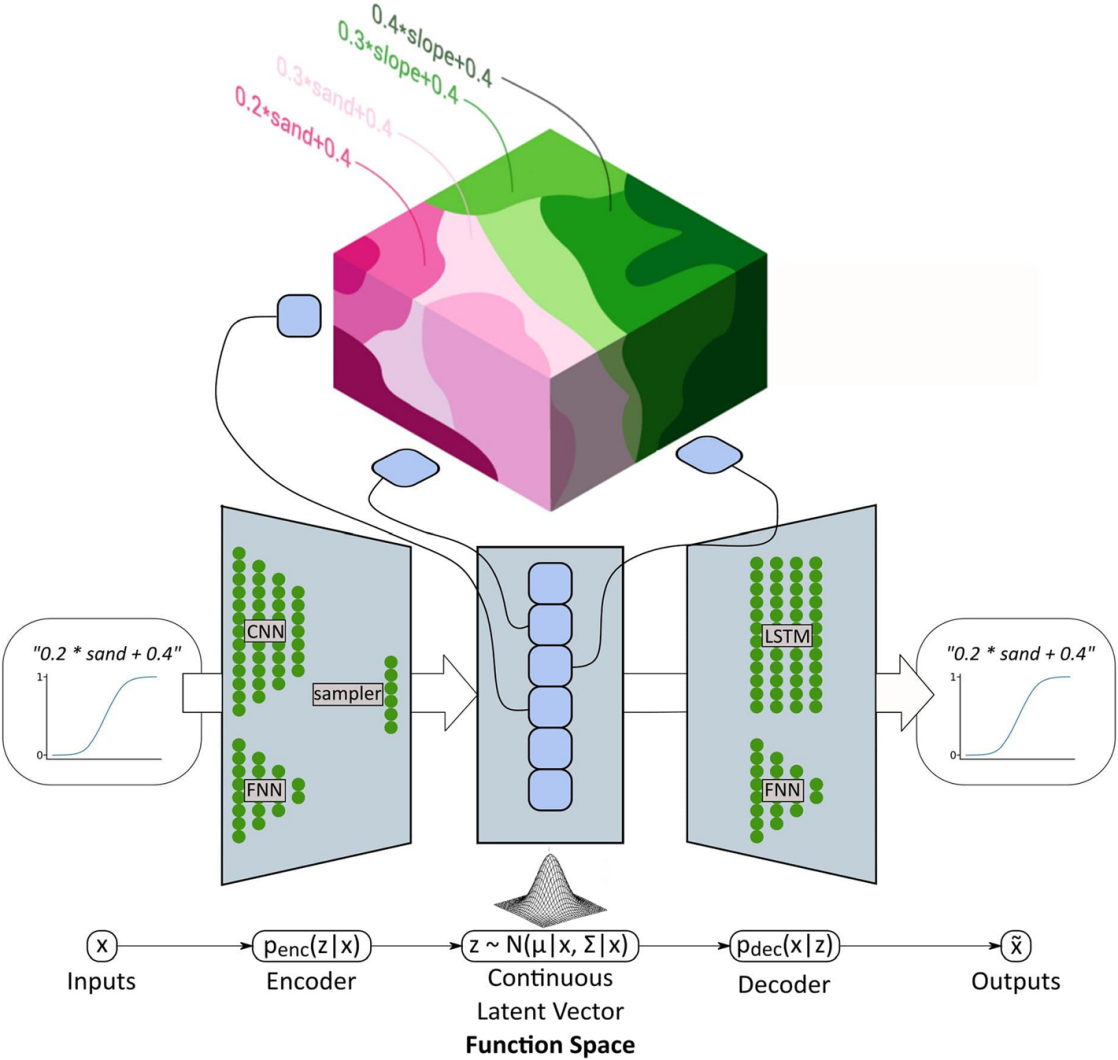
A simplified depiction of the FSO variational autoencoder with an example function that gets transferred to the function space and reconstructed to its original form. The inputs are transferred to the function space using the encoder network. The function space representation is then passed through the decoder network to reconstruct the inputs. The function space is a six‐dimensional continuous vector space with a Gaussian distribution.

One type of generative models often used in NLP is autoencoders (Le Cun and Fogelman‐Soulié, 1987). Autoencoders consist of two neural networks, an encoder network which maps the input to a continuous vector representation and a decoder network which reconstructs the encoded input from the continuous vector representation (see Figure 2). A main advantage of using an autoencoder is the resulting low dimensional continuous vector representation of the inputs. This continuous vector representation is called the *latent representation* or *latent space* of the input information. After training an autoencoder to correctly encode and decode the information of a set of strings, the decoder can be used to generate strings from the latent space.

When left unconstrained, the latent space could potentially be sparse, meaning that large areas within the space would not produce any valid functions. Consequently, it is necessary to constrain it. To include a constraint on the latent space, we use a variational autoencoder (VAE) architecture (Kingma and Welling, 2013). VAEs add stochasticity to the latent space, which results in a latent representation that is more robust to small variations. Furthermore, it enforces a certain distributional behavior (usually Gaussian) onto the space by adding a penalty term. A definition of the VAE architecture is given in the supporting information (Text S1). A detailed definition and derivation of VAEs and their properties can be found in Kingma and Welling (2013).

A simplified representation of the FSO VAE is shown in Figure 2. The encoder consists of a combination of word embeddings (Mikolov et al., 2013), convolutional layers (CNN) (LeCun et al., 1989) and feedforward neural network (FNN) layers (White & Rosenblatt, 1963) with SELU (scaled exponential linear unit) activation functions (Klambauer et al., 2017). The decoder is a combination of FNN layers with SELU activation functions and a long short‐term memory (LSTM) network (Hochreiter & Schmidhuber, 1997). The chosen architecture for the encoding and decoding of function strings was inspired by an architecture developed by Gan et al. (2018). The additional encoding and decoding of the parameter distributions aim to further condition the latent space to also include the information about the resulting parameter distribution in the latent space. A detailed description of the FSO VAE is given in the supporting information (Text S2 and Figure S1).

To incorporate both semantic and parameter distribution information in the autoencoder, two kinds of inputs/outputs are used for training: the transfer function strings and the parameter distribution resulting from that transfer function. The transfer function is given as a vector of symbols, for example, “sand,” “+,” and “slope.” The dimension of this vector equals the maximum length of a transfer function created by the CFG. The parameter distribution is given as a numeric vector containing the 0.1 to 0.9 quantiles in 0.1 steps and is estimated from the spatial predictors of the catchment. The FSO VAE encodes this information into a six‐dimensional numerical space that we call *Function Space*.

The FSO VAE is trained to minimize three type of losses: (1) the cross‐entropy loss resulting from the reconstruction of functions strings, (2) the mean square error of the parameter distribution reconstruction, and (3) the Kullback‐Leibler divergence (Kullback & Leibler, 1951) between the function space and a multivariate normal distribution. They are weighted with factors to balance their importance during training. A detailed description of the loss function is given in the supporting information (Text S2).

After training, the VAE is able to reconstruct the input data, and the decoder can generate a function from every point in function space, which has the same properties as defined by the CFG. Since the VAE is able to reconstruct function strings and their parameter distributions, the distance in the function space reflects both semantic closeness and closeness in the resulting parameter distributions. This leads to a reasonable approximation of the closeness of loss functions, making it an adequate continuous space for optimization.

#### 2.2.3 Normalization

To enable the unbiased estimation of universally applicable transfer functions, a cascade of scaling is necessary to:

- make the spatial predictors of the catchment comparable,
- make the transfer functions usable in areas where the range of spatial predictors is outside of the observed range of the catchment used for transfer function estimation, and
- be able to predict a certain model parameter in the correct range of its feasible values.

Scaling for any values *x* to an arbitrary interval [*a*,*b*] is done in FSO by applying the min‐max scaling function: $x_{\left[a,b\right]}=a+\frac{\left(x-\min\left(x\right)\right)\left(b-a\right)}{\max\left(x\right)-\min\left(x\right)}$.

To be able to compare the information from multiple spatial predictors of the catchment, they are scaled to the interval [0,1] (i.e., data normalization). An issue of trying to find parameter transfer functions that are universally applicable is the dependency on the scale of the catchment that was used to derive it. To avoid this restriction, the scaling to the interval [0,1] is done by using their physically possible or reasonable minimum and maximum values. For example, all slope values are scaled from the interval [0,90] to [0,1].

To be able to estimate the parameters in their corresponding scale, the values resulting from applying the transfer functions are rescaled to the parameter bounds. The parameter bounds need to be chosen for each parameter and should reflect the values in which the parameters have any (physical) meaning. This allows for global scale application.

#### 2.2.4 Optimization in Function Space (FSO)

**Figure 3:**
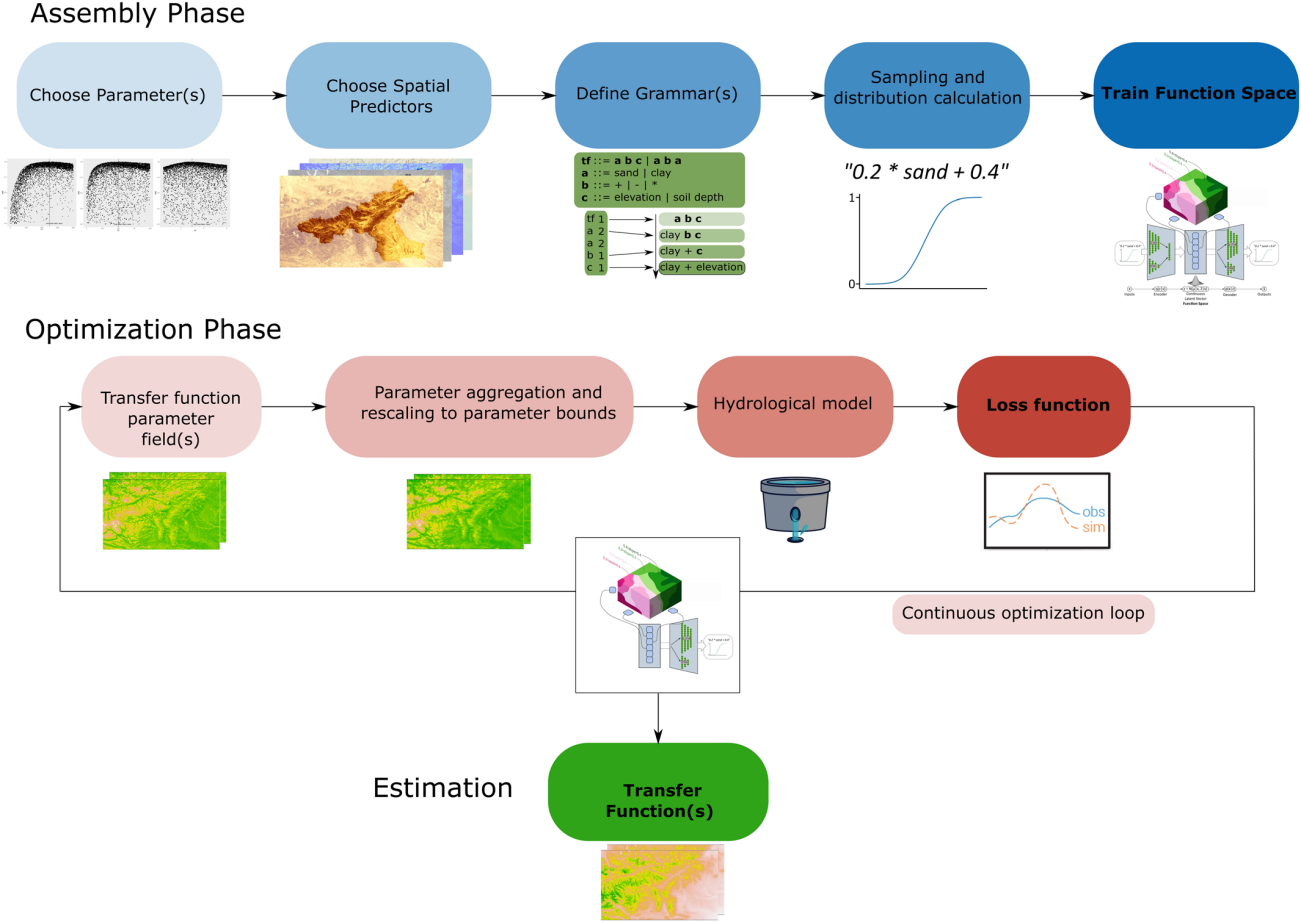
The function space optimization workflow. It consists of two phases, the assembly phase with all the necessary steps to train the FSO VAE and the optimization phase in which a continuous optimization algorithm is used to optimize in function space. The decoder part of the VAE is used in the optimization loop to generate new functions from function space.

The full workflow of FSO is shown in Figure 3. It consists of two main parts: the assembly phase and the optimization phase. The steps of the assembly phase were already described in sections 2.2.1–2.2.3. It includes the selection of parameters, the selection of spatial predictors, the CFG definition, and the training of the VAE.

The optimization phase of the FSO is a fully automatic procedure that uses the text generating VAE which was trained in the assembly phase. It searches for the optimal point in the function space (i.e., transfer function[s]) to minimize the loss function. In each iteration a new function is generated from the function space, which is used to produce a parameter field. This new parameter field is used in the hydrological model and results in a loss function output. After a previously defined number of iterations, the function with minimum loss is chosen as the estimation for the parameter transfer function.

In general, any continuous optimization algorithm can be applied in the optimization phase. We experimented with three commonly used algorithms: Genetic Algorithm (Taylor, 1994), Dynamically Dimensioned Search (DDS; Tolson & Shoemaker, 2007), and the Particle Swarm Optimization (Kennedy & Eberhart, n.d.). All of them were able to solve the given optimization problem equally well. Our tests showed that the DDS performed slightly more consistently than the other two. Consequently, we decided on using the DDS for the optimization in function space.

FSO can optimize multiple parameters at the same time. This can be done by optimizing multiple function spaces. Since each function is represented as a six‐dimensional continuous vector in function space, optimizing two transfer functions results in a 12‐dimensional continuous optimization problem.

## 3 Case Study

To test whether FSO is able to sufficiently approximate transfer functions, we conducted a test in a virtual reality setting. This case study applies FSO on a parsimonious distributed model using synthetic runoff data.

### 3.1 Mur Catchment

**Figure 4:**
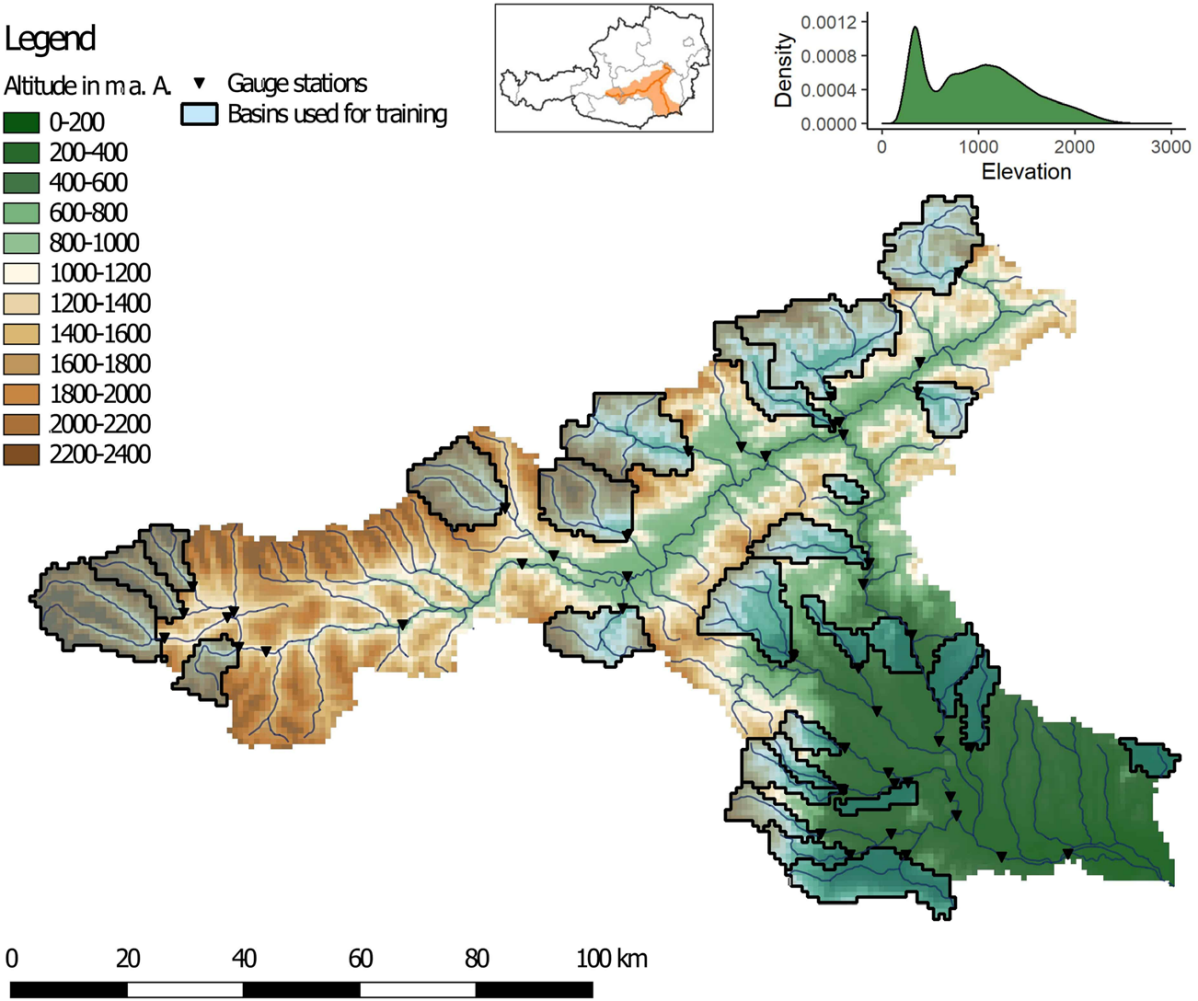
The digital elevation model of the Mur catchment on a 2 km grid. The 27 headwater basins used for training FSO are shown in blue. The top right part of the figure depicts the catchments locations in Austria and its elevation distribution.

The case study was performed using hydrological, meteorological, climate, and geographic data from the Mur catchment (see Figure 4), which is located in the southeastern part of Austria and has an area of 10,420 km^2^. For testing FSO, we intended to use data from a catchment with a wide range in physical properties to explore its applicability on a large scale. As the Mur consists of high alpine areas in the north and significantly lower areas in the south and very diverse geology, it fulfills this condition. Another reason for selecting this catchment was to compare the further development in the regionalization method with the work by Klotz et al. (2017).

The 250 m gridded geophysical properties used in this case study are height above sea level (*elevation*), slope (*slope*), height above nearest drainage (*hand*), percentage of clay (*clay*), percentage of sand (*sand*), soil depth (*bdim*), the enhanced vegetation index (*evi*), and a noise layer (*noise*). Topographic properties (*elevation*, *slope*, and *hand*) were calculated from a digital elevation model obtained from Rechenraum (2012), and soil information (*clay*, *sand*, and *bdim*) was obtained from SoilGrids (Hengl et al., 2017). SoilGrids is a system for digital soil mapping using state‐of‐the‐art machine learning. The *evi* layer was derived by averaging an *evi* time series for the years 2000–2017 from Didan (2015). For further information about the enhanced vegetation index refer to Huete et al. (2002). In addition to the observation data, we generated a noise layer (*noise*) that consists of values sampled from a uniform distribution over the interval [0, 1]. The *noise* layer was created for further testing FSO by providing irrelevant information as a possible predictor.

All geophysical properties are strongly correlated with an overall mean absolute correlation coefficient of 0.55 (these values do not include *noise*). Very high correlation coefficients could be observed for clay/sand (−0.95), clay/elevation (−0.83), and elevation/bdim (−0.82), and the lowest was observed for evi/hand (−0.35).

The meteorological data used in this case study are air temperature and precipitation from the INCA analysis (Haiden et al., 2011, 2014). The potential evapotranspiration was computed using the Thornthwaite equation (Thornthwaite & Mather, 1957).

To evaluate the predictive capability of the algorithm, two data splits were applied. First, we split the time series and used the period January 2003 to August 2009 for training and the years September 2009 to December 2012 for testing. Second, we split the basins and used 27 headwater basins (marked as blue in Figure 4) for training and 95 basins for testing. Thereby, we estimate not only the ability to predict an independent time period but also the ability to predict runoff in ungauged basins.

### 3.2 Distributed GR4J

**Figure 5:**
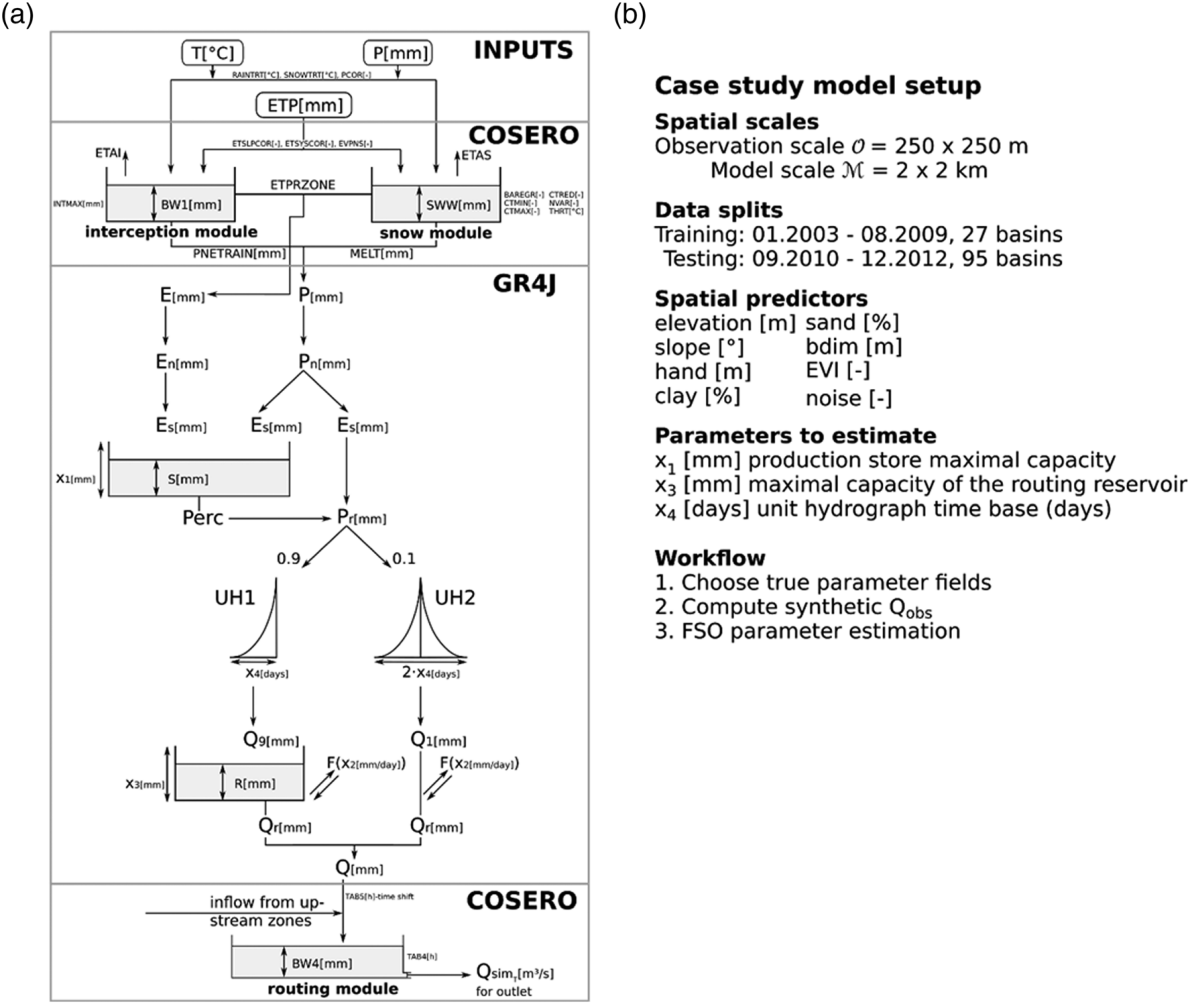
(a) Model structure of d‐GR4J, showing implemented parts from COSERO and GR4J. (b) Overview of the case study model setup, including spatial scales, data splits and spatial predictors used for the simulations and a summary of estimated parameters and workflow steps.

For testing purposes, the parsimonious hydrological model GR4J (Perrin et al., 2003) was chosen. GR4J is a lumped hydrological model for predicting daily mean runoff. It is a simple four‐parameter model, consisting of two storages and two unit hydrographs (Sherman, 1932). The GR4J model structure can be seen in the middle part of Figure 5a. To implement GR4J as a distributed model and to include snow and interception processes, we combined it with the routing, interception, and snow module from the COSERO (Continuous SEmidistributed RunOff model) model. It is a HBV‐type model which was developed by Nachtnebel et al. (1993) and was applied in lumped and semidistributed (Kling et al., 2015; Stanzel et al., 2008) and in distributed settings (Frey & Holzmann, 2015; Herrnegger et al., 2012, 2018; Kling et al., 2006; Kling & Nachtnebel, 2009; Wesemann et al., 2018). We will refer to this extended version of GR4J as d‐GR4J. The complete d‐GR4J model structure can be seen in Figure 5a.

GR4J consists of four parameters: X_1_ production store maximal capacity (mm), X_2_ catchment water exchange coefficient (mm/day), X_3_ 1 day maximal capacity of the routing reservoir (mm), and X_4_ unit hydrograph time base (days). A more detailed description of the GR4J model is given by Perrin et al. (2003). All parameters from the COSERO part of d‐GR4J were taken from previous calibration of COSERO for the Mur catchment, leaving only the four GR4J parameters to be optimized.

In order to demonstrate that d‐GR4J is generally able to adequately describe catchment hydrological processes, we performed an initial conventional parameter optimization against observed discharge data, using the DDS algorithm. To further investigate whether the GR4J parameters are a reasonable choice for the parameter transfer function estimation, we examined their sensitivity with a Monte Carlo parameter simulation and a global parameter sensitivity estimation using the Fourier amplitude sensitivity test (FAST) (Cukier et al., 1978) which was already applied on multiple hydrological models (e.g., Francos et al., 2003; Y. Gan et al., 2014; Ratto et al., 2001; Reusser et al., 2011). While a Monte Carlo simulation provides useful insight, it does not provide a quantitative sensitivity estimation (Wang, 2012). FAST estimates the fractional contribution of individual parameters to the variance of the output and therefore quantifies the sensitivity of individual parameters.

### 3.3 Design of a “Virtual Hydrological Reality”

Prior to real‐world applications, it is necessary to test the principle functionality of FSO under more controlled conditions to avoid possible sources of errors such as measurement errors, wrong assumptions for model parameters, or missing spatial predictors. We used real observation data for the catchment properties but generated the discharge values synthetically, using a priori defined “true” transfer functions. These true transfer functions are used to generate d‐GR4J parameter fields and, with observed climate data as input, time series of discharge data for each subbasins. In the following, these discharge data were treated as observations from which the true transfer functions were reestimated.

Our objective is to reestimate transfer functions for three GR4J parameters: X_1_, X_3_, and X_4_. The parameter X2 allows for water inflow or outflow of the basin. Because X2 is not reflected in precipitation or discharge, it was set to 0. Hence, we assumed that there is no unobserved water inflow or outflow in the catchment.

**Figure 6:**
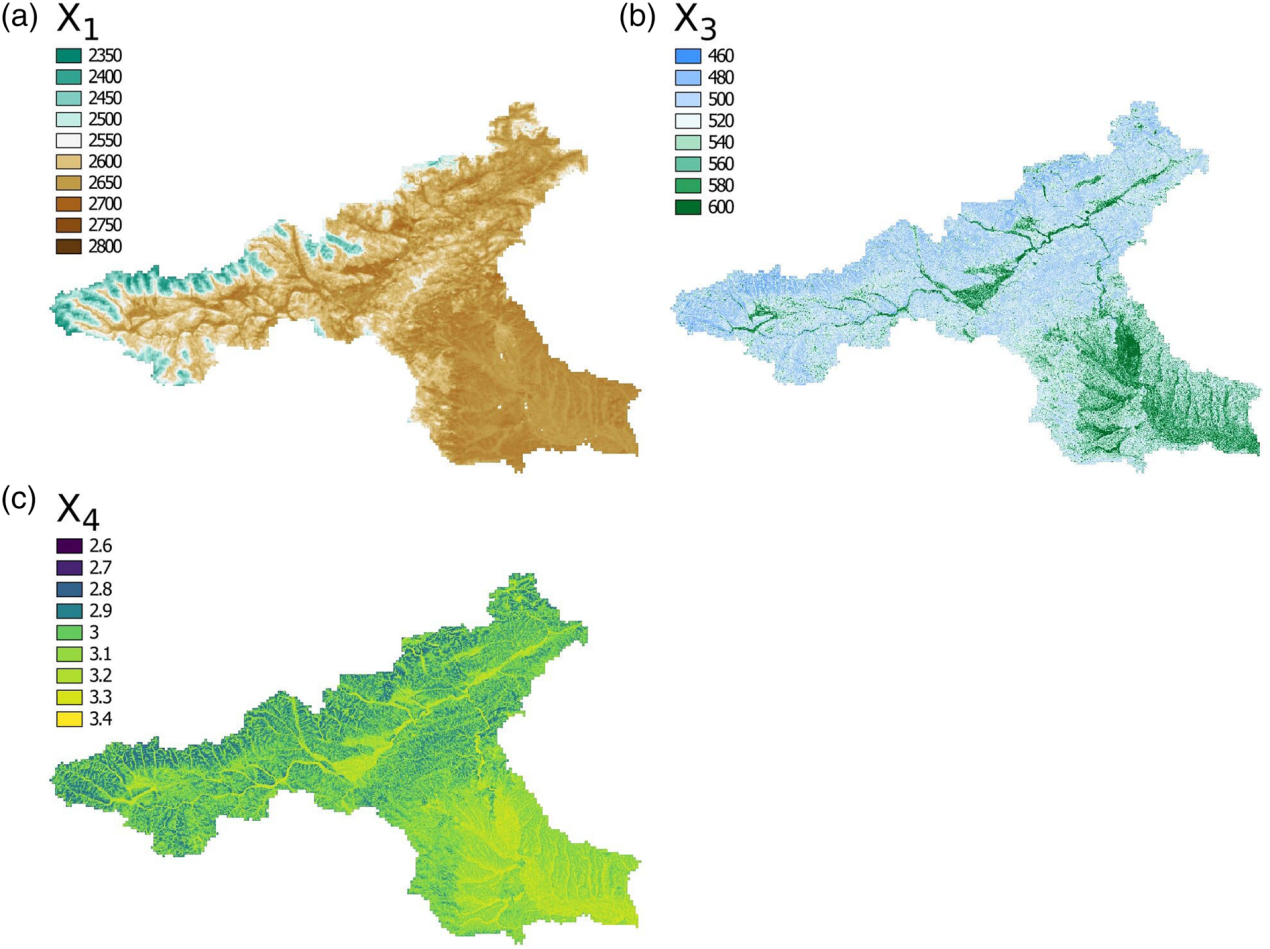
True parameter field for the three d‐GR4J parameters (a) X1, (b) X3, and (c) X4.

We chose the true underlying transfer functions that use one or two different spatial predictors. The transfer functions for the three parameters are chosen to reflect the possible physical interpretation of the parameters, for example, large values of *evi* and soil depth (*bdim*) result in a large production store. Their resulting parameter fields are shown in Figures 6a–6c. The chosen true transfer functions are as follows:

(4) $$X_{1}=0.5+\mathrm{evi}\cdot 1.5+\exp\left(\text{bdim}\right)\cdot 0.9,$$

(5) $$X_{3}=-1.3-\log\left(\text{slope}\right),$$

(6) $$X_{4}=-1.5-\log\left(\text{hand}\right)\cdot 0.2-\text{slope}\cdot 1.5.$$

The CFG for the case study is chosen to be complex, to create a large search space. It included multiple recursive nonterminals, exp/log functions, power functions, reciprocal functions, and linear combinations. Additionally, to include the search for the global parameters *β*, the numerical values −1.5 to 1.5 in steps of 0.1 are added as terminal symbols. Since all spatial predictors are in the interval [0, 1], the range [−1.5, 1.5] for numerical values should include enough complexity to simulate a real task. Nevertheless, in a real‐world application it might be helpful to increase the range of numerical values and allow for a wider range of functions (e.g., sin and tan), which would only increase computation time in the assembly phase of FSO and not for the optimization. The complete CFG used in the case study is shown in the supporting information (Figure S2). The three true transfer functions can be generated from the CFG and are therefore included in our possible realm of transfer functions.

From this CFG we sampled 5 million unique functions. We used 80% as the training set and 20% as the validation set for the VAE. The minimum validation loss was reached after 132 epochs of training the VAE with a batch size of 1,000.

After selecting the true transfer functions, the resulting parameter fields are used to produce a synthetic discharge time series using d‐GR4J on a 2 km grid. Also, the internal states of the storage S and R for each grid and each time step are calculated. An overview of the case study model setup is shown in Figure 5b.

### 3.4 Applying FSO

The performance of FSO is strongly dependent on the choice of the loss function *f*_*loss*_. Therefore, two different tests were conducted using:

1. a single‐objective criterion, with loss only dependent on discharge, and
2. a multivariate objective criterion, with loss dependent on discharge and state “observations.”

For both tests, the optimization procedure was repeated five times to evaluate the variations in the retrieval process, while a maximum number of 3,000 iterations for the DDS global optimization algorithm was chosen.

We applied the multicriteria FSO tests (Tests 2 and 3) using S, R, and both simultaneously. For brevity, we here present only the optimization using the time series of state S. The results for the additional tests can be found in the supporting information (Figures S6–S9).

#### 3.4.1 Test 1: Single‐Objective Criteria

Test 1 focuses on estimating transfer functions by considering a loss that is only dependent on the predicted and observed discharge. The loss function is formulated similar to the Nash‐Sutcliffe efficiency $\mathrm{NSE}=\frac{\sum_{\mathrm{it}=1}^{T}{\left(Q_{m}\left[t\right]-Q_{o}\left[t\right]\right)}^{2}}{\sum_{\mathrm{it}=1}^{T}{\left(Q_{m}\left[t\right]-\bar{Q_{o}}\right)}^{2}}$ (Nash & Sutcliffe, 1970), using a weighted mean NSE value of the form

(7) $${\mathrm{NSE}}_{\mathrm{wm}}=\frac{\sum_{i=1}^{m}w_{i}\mathrm{NSE}\left(Q_{s,i},Q_{p,i}\right)}{\sum_{i=1}^{m}w_{i}},$$

with the weights *w*_*i*_ = 1 − *NSE*(*Q*_*s*,*i*_, *Q*_*p*,*i*_) for all *i* ∈ {1, …, *m*}, which is a weighted arithmetic mean over m basins. *Q*_*s*,*i*_ and *Q*_*p*,*i*_ are the synthetic and predicted time series of discharge for basin *i*, respectively. By using this form of averaging, the basins with the lowest NSE values get the highest weight, while basins with NSE close to 1 become unimportant. This forces the optimization procedure to estimate transfer functions that operate equally well in all catchments.

Finally, an additional penalty term is added to the loss function to reduce the likelihood of overfitting. This term penalizes for the transfer function length, that is, the number of symbols used in a function, which can be interpreted as the function complexity. We defined it as *loss*_*size*_ = transfer function length · 0.001 resulting in

(8) $$f_{\text{loss}}=-{\mathrm{NSE}}_{\mathrm{wm}}+{\text{loss}}_{\text{size}}.$$

#### 3.4.2 Test 2: Multivariate Objective Criteria Using NSE

To implement a multicriteria optimization in FSO, we adapt *f*_*loss*_ to include the loss from additional sources. In Test 2, we assume the existence of an additional observation of time series of our GR4J system states S (production store) and R (routing store) and define a multicriteria weighted mean NSE as

(9) $${\mathrm{NSE}}_{\mathrm{wm}\_\text{multi}}=\frac{\sum_{i=1}^{n}w_{i}{\mathrm{NSE}}_{\text{multi},i}}{\sum_{i=1}^{n}w_{i}},$$

with *NSE*_*multi*,*i*_ = 0.5 · (*NSE*(Q_s,i_, Q_p,i_)+NSE(State_s,i_, State_p,i_)) and the weights *w*_*i*_ = 1 − *NSE*_*multi*,*i*_ *for all i* ∈ {1, …, *n*}. State_s,i_ and State_p,i_ are the mean synthetic and mean predicted time series of model states for basin *i*, respectively. *NSE*_*multi*,*i*_ is the arithmetic mean of the NSE values used in the multicriteria objective of basin *i*. Hence, when optimizing discharge and a model state, it results in using the mean of two NSE values. Using Equation 8 again, we can thus define our multicriteria loss function:

(10) $$f_{\text{loss}}=-{\mathrm{NSE}}_{wm_{\text{multi}}}+{\text{loss}}_{\text{size}}.$$

#### 3.4.3 Test 3: Multivariate Objective Criteria Using SPAEF

Similar to Test 2, we adapt *f*_*loss*_ to include the loss of additional observations of system states in this test. However, instead of intepreting the system states as independent time series of states for each basin, model states are integrated as a single time series of gridded maps. This interpretation allows us to use the SPAEF (SPAtial Efficiency metric) (Demirel, Koch, et al., 2018; Demirel, Mai, et al., 2018) to compute a loss from the spatial pattern of the states for each time step. Inspired by the Kling‐Gupta efficiency (KGE) (Gupta et al., 2009), SPAEF combines correlation, fraction of the coefficients of variation, and the histogram intersection (Swain & Ballard, 1991). The SPAEF for comparing synthetic and predicted model states of one timestep can be written as

(11) $$\text{SPAEF}=1-\sqrt{{\left(\alpha -1\right)}^{2}+{\left(\beta -1\right)}^{2}+{\left(\gamma -1\right)}^{2}},$$

$$\alpha =\rho \left({\text{State}}_{s},{\text{State}}_{p}\right),\;\beta =\left(\frac{\sigma_{{\text{State}}_{p}}}{\mu_{{\text{State}}_{p}}}\right)/\left(\frac{\sigma_{{\text{State}}_{s}}}{\mu_{{\text{State}}_{s}}}\right)\;\text{and}\;\gamma =\frac{\sum_{j=1}^{n}\min\left(K_{j},L_{j}\right)}{\sum_{j=1}^{n}K_{j}},\;$$

where *α* is the pearson correlation coefficient, *β* the fraction of coefficients of variation of predicted and synthetic states, and *γ* the histogram intersection of the two corresponding histograms *K* and *L* with *n* bins. Similar to the NSE and KGE, the resulting SPAEF values are in the range [−∞, 1]. SPAEF can also be used to compare spatial patterns of two maps with different units, if the observed and predicted values are standardized before computing the histogram intersection (Koch et al., 2018). This allows application in situations where spatial observations do not explicitly represent model states but are associated with them. To simulate such a case, both state maps were standardized before computing the histogram intersection.

The loss function for this test can thus be defined as follows:

(12) $$f_{\text{loss}}=-\frac{1}{2}\left({\mathrm{NSE}}_{\mathrm{wm}}+{\text{SPAEF}}_{m}\right)+{\text{loss}}_{\text{size}},$$

where *NSE*_*wm*_is the weighted mean basin, *SPAEF*_*m*_ the mean SPAEF of all time steps excluding spin up time, and *loss*_*size*_ the penalty for function complexity as defined in section 3.4.1. In case of multiple states, *SPAEF*_*m*_ is equal to the overall mean of both states.

## 4 Results

### 4.1 Function Space

First, we will illustrate the properties of the FSO function space by analyzing the generated functions and resulting parameter distributions when moving on a straight line between two points in the space. As distance in function space should reflect not only semantic closeness but also closeness in their resulting parameter distributions, we expect to see a gradual change in both properties in this linear interpolation, thus indicating an appropriate searchability.

**Figure 7:**
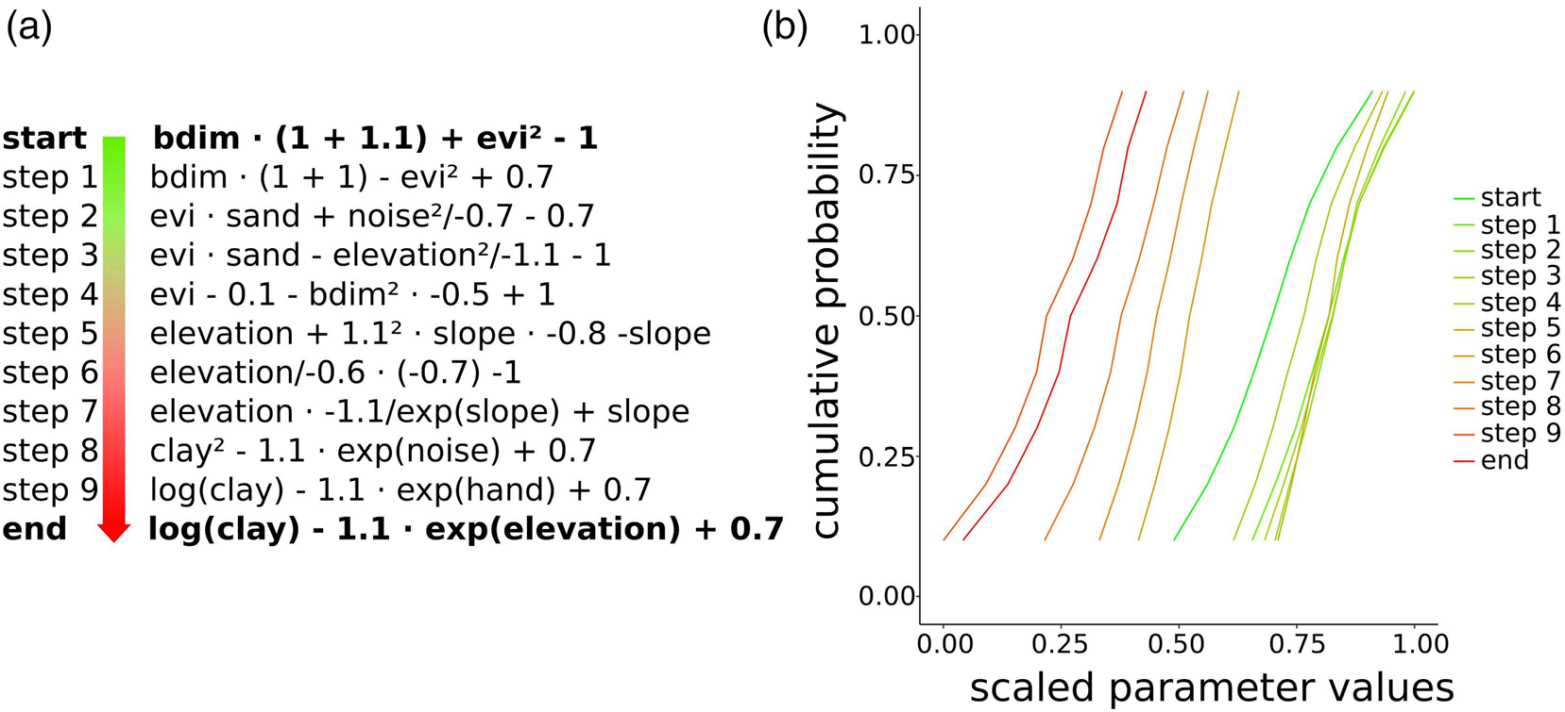
A linear interpolation in function space. (a) Functions generated along a straight line between start and end points in function space. (b) Corresponding quantiles of scaled parameter values. Parameter values are scaled by min‐max scaling using the range of all parameter values in the training data.

We chose two random points in function space, *F*_1_ and *F*_2_, and linearly interpolate between them *F*(*w*) = *w* · *F*_1_+(1 − *w*) *F*_2_, with weights *w* ranging from 0.1 to 0.9 with 0.1 steps between them. Hence, we produce new functions from FS while moving on a line between *F*_1_ and *F*_2_ on every tenth of the way. The corresponding functions are shown in Figure 7. Figure 7a shows the generated function strings. The further away we move from the starting point, the more function strings resemble the end point function. Figure 7b shows the corresponding parameter distributions resulting for all functions in this linear interpolation. Here we observe that functions closer to each other in FS are also closer in terms of produced parameter distribution.

### 4.2 Global Sensitivity and Model Performance

**Figure 8:**
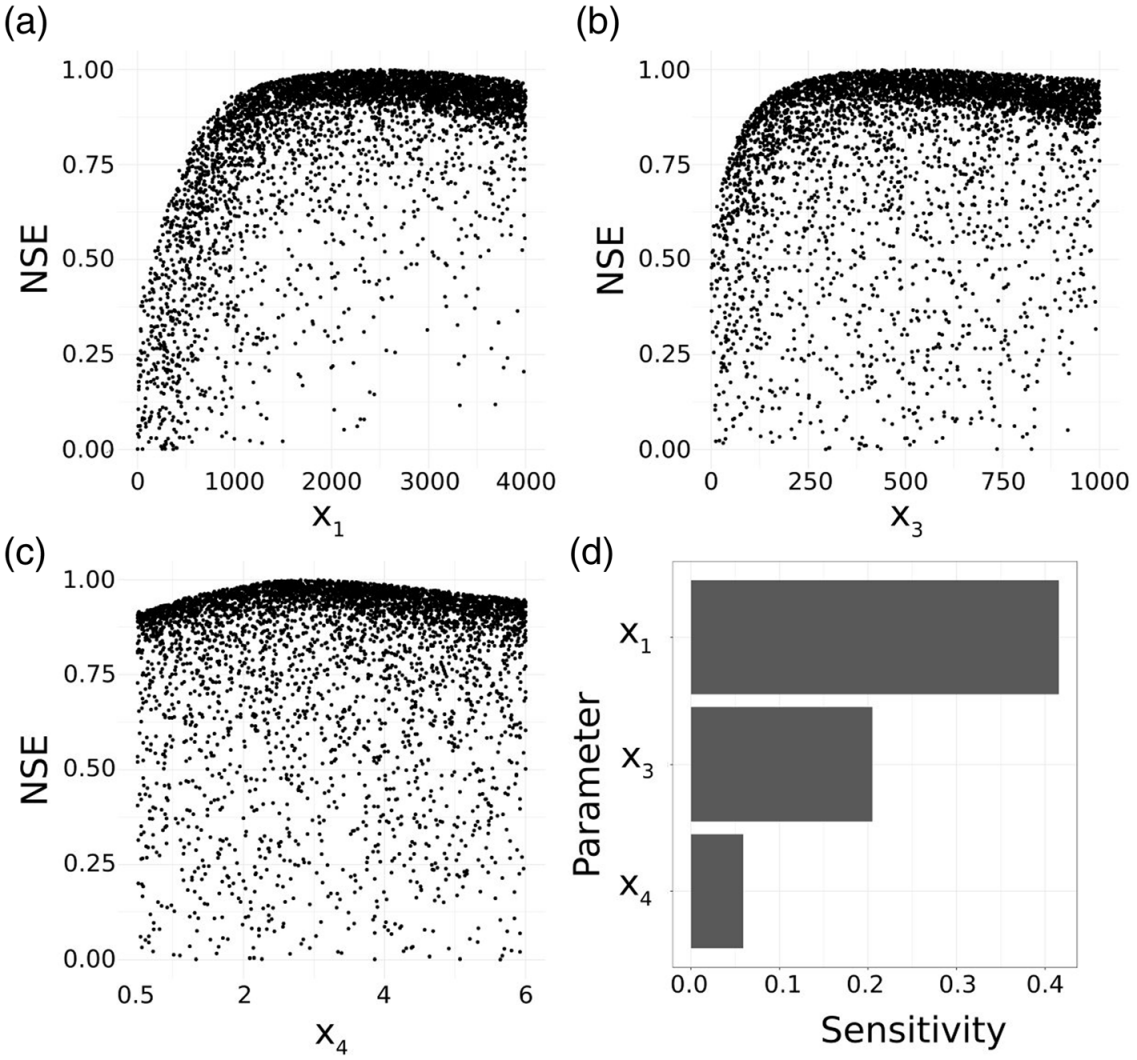
Parameter sensitivity of one subbasin of the Mur catchment. (a–c) Parameter response surface for all three d‐GR4J parameters with NSE values cut‐off below 0. (d) Results from the FAST sensitivity analysis showing the percentage contribution of individual parameters to the variance of the output.

The conventional optimization on real observation data showed that d‐GR4J is able to map observed runoff dynamics resulting in a mean basin NSE of 0.78 with a minimum NSE of 0.57 and a maximum NSE of 0.91. Therefore, we can assume a general ability of d‐GR4J to model rainfall‐runoff processes in the study catchment. A relevant property of d‐GR4J regarding the estimation of transfer functions is the parameter sensitivity. Figures 8a–8c show the parameter response surfaces resulting from the Monte Carlo simulations. We can see peaks for all three parameters and a clearly defined response surface. Figure 8d shows the results from the parameter sensitivity analysis using FAST. X1 has the highest sensitivity with ~40% contribution to the output variance, while X3 and X4 are less sensitive with ~20% and ~5%, respectively.

### 4.3 Test 1: Single‐Criteria FSO Using NSE for Q

**Figure 9:**
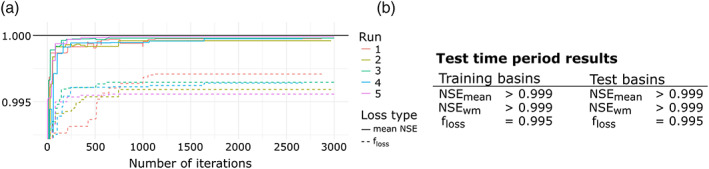
(a) Single‐criteria FSO training results for all five runs. (b) Summary of performance during test time period for training and test basins.

Figure 9a shows the training performance of all five single‐criteria FSO optimization runs (see section 3.4), that is, results of the training basins for the training time period. The different runs are distinguished by their color. The two line types show the mean NSE (solid) and *f*_*loss*_ (dashed) as defined in Equation 5. It is clearly visible that the performance of FSO is stable, with a spread of mean basin NSE of less than 0.001. Furthermore, all runs arrive at a solution with NSE > 0.995 in less than 250 iterations.

For brevity, we will show the detailed results of one run only, which describes the general behavior of all runs. Figure 9b shows the model performance in the test time period. It is notable that we can observe the same quality of results for training (“gauged”) and test (“ungauged”) basins and both are close to the possible maximum, with an NSE of >0.999. Naturally, such high NSE values are only possible in a synthetic setting in which we use the correct model and error free observation data.

The comparison between true *f*_*tf*_ and single‐criteria FSO estimated *f*_*tf*_ can be seen in Table 1. FSO was able to predict the correct spatial predictor for X3 and one of the two spatial predictors of X1. Examining the true *f*_*tf*_ for X1, we can note that due to the exponent, *bdim* has a larger influence on the pattern of parameter values and is therefore much easier to find than *evi*. This is due to a greater variability that is more likely to produce unique patterns: exp (bdim) has a standard deviation of 0.337, while evi*1.5 has a standard deviation of 0.094.

**Table 1:** Comparison of the True *f*_*tf*_ and the Single‐Criteria FSO Estimated *f*_*tf*_

Parameter	True *f*_*tf*_	FSO estimated *f*_*tf*_
X1	0.5+*evi* · 1.5+exp(**bdim**) · 0.9	1.1+hand · 1.3 − elevation+exp(**bdim**)
X3	−1.3 − log(**slope**)	1.5 − **slope**/0.2
X4	−1.5 − log(hand) · 0.2 − slope · 1.5	0.7+log(clay)

**Figure 10:**
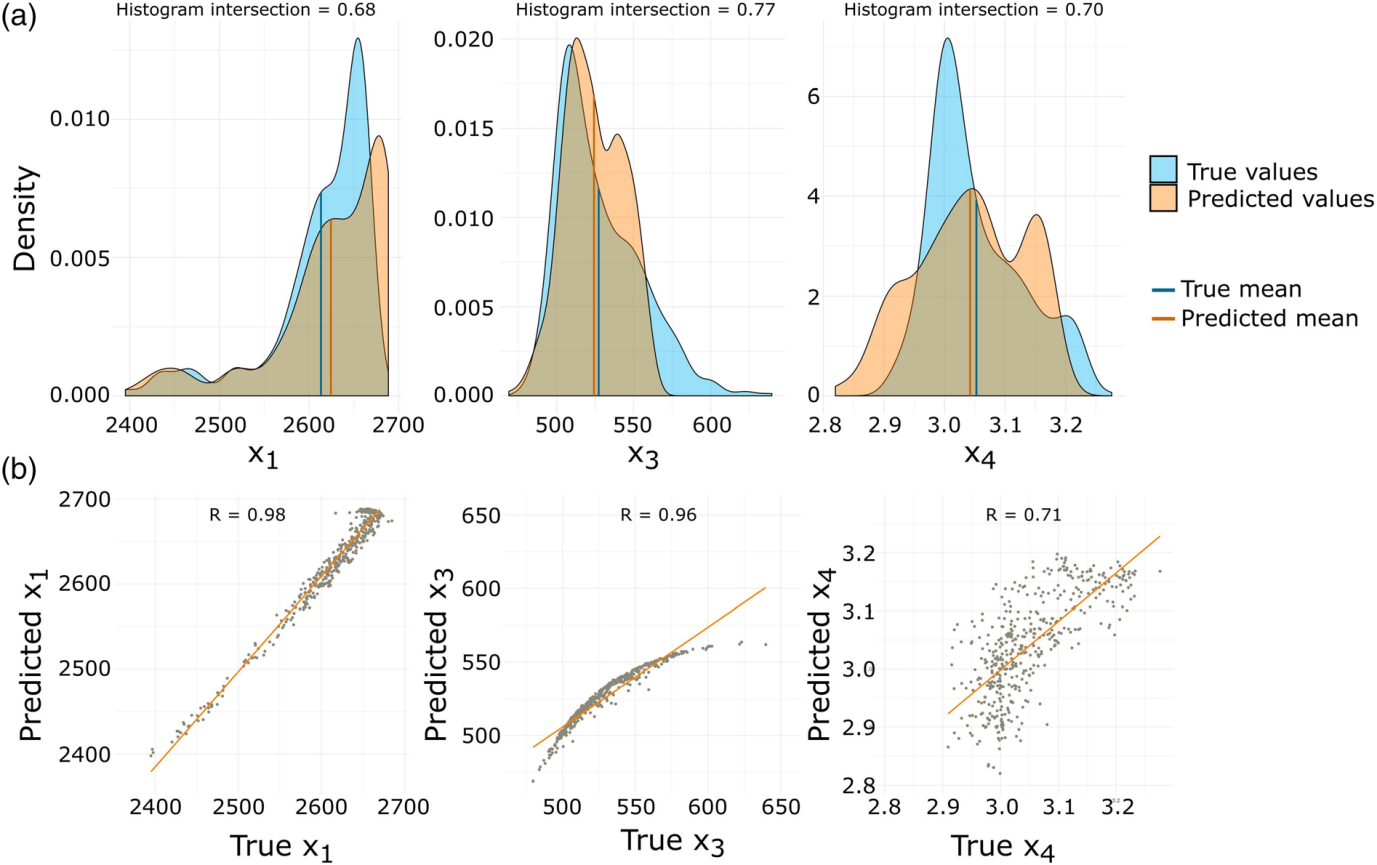
Single‐criteria FSO results for all three optimized d‐GR4J parameters on the 2 km model scale. (a) Estimated and true parameter densities with their mean values and corresponding histogram intersection. (b) Scatterplots of true versus estimated parameters and fitted linear model with Pearson correlation coefficients.

Figure 10 shows the estimated and true parameter distributions and scatterplots for all three optimized parameters. The means of the predicted parameter values are nearly the same for all three parameters, with X1 having the largest difference (10.88 or 3.67% of the total parameter value range). We can see that for X1 and X3, the parameter distributions of true and predicted *f*_*tf*_ are nearly identical for smaller values and more diverging for larger values. Figure 10b shows the linear relationship corresponding to a correlation of 0.98 for the predicted and true values of X1. Here, the predicted values of X3 seem to have a nonlinear relationship but nevertheless a correlation of 0.96. For X4 we see a larger difference in the parameter density (correlation of 0.71), but it is notable that the mean value is nearly identical.

A comparison of the estimated parameter fields and the true parameter fields is shown in the supporting information (Figure S3).

### 4.4 Test 2: Multicriteria FSO Using NSE for Q and S

**Figure 11:**
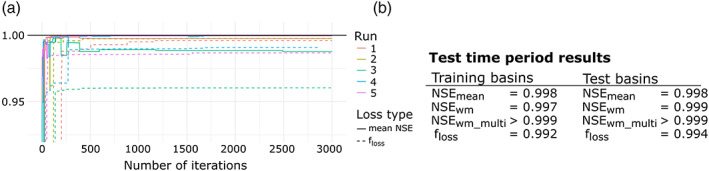
(a) Training results for all five multicriteria FSO runs using NSE for Q and S. (b) **S**ummary of performance during test time period for training and test basins.

Figure 11a shows the training performance of all five multicriteria FSO optimization runs using the d‐GR4J state S as additional optimization criteria. Therefore, the multivariate objective function consists of the NSE evaluated against Q and system state S. The state S is only controlled by the parameter X1; hence, we expect an improvement in estimating *f*_*tf*_ for X1. Compared to the single‐criteria FSO, multicriteria FSO has a slightly increased variance in *f*_*loss*_. It is still very stable in regard to the mean NSE. Only Run 3 is somewhat different with a training mean NSE of 0.988.

For brevity, we will show the detailed results of one run only, which describes the general behavior of all runs. Figure 11b shows the model performance in the test time period. It is notable that we can observe the same quality of results for gauged and ungauged basins with both having an NSE ≥0.998.

The comparison between true *f*_*tf*_ and multicriteria FSO estimated *f*_*tf*_ can be seen in Table 2. FSO was able to predict both spatial predictors of X1 correctly. The slope was included in the estimated *f*_*tf*_ of X3 and X4 correctly; otherwise, the structure of the functions is different.

**Table 2:** Comparison of the True *f*_*tf*_ and Estimated *f*_*tf*_ by the Multicriteria FSO Using NSE for Q and S

Parameter	True *f*_*tf*_	FSO estimated *f*_*tf*_
X1	0.5+**evi** · 1.5+exp(**bdim**) · 0.9	0.8+exp(**evi**)+**bdim**2/0.8
X3	−1.3 − log(**slope**)	log(bdim)+**slope**
X4	−1.5 − log(hand) · 0.2 − **slope** · 1.5	log(clay) − (bdim · log(**slope**)+**slope**)

**Figure 12:**
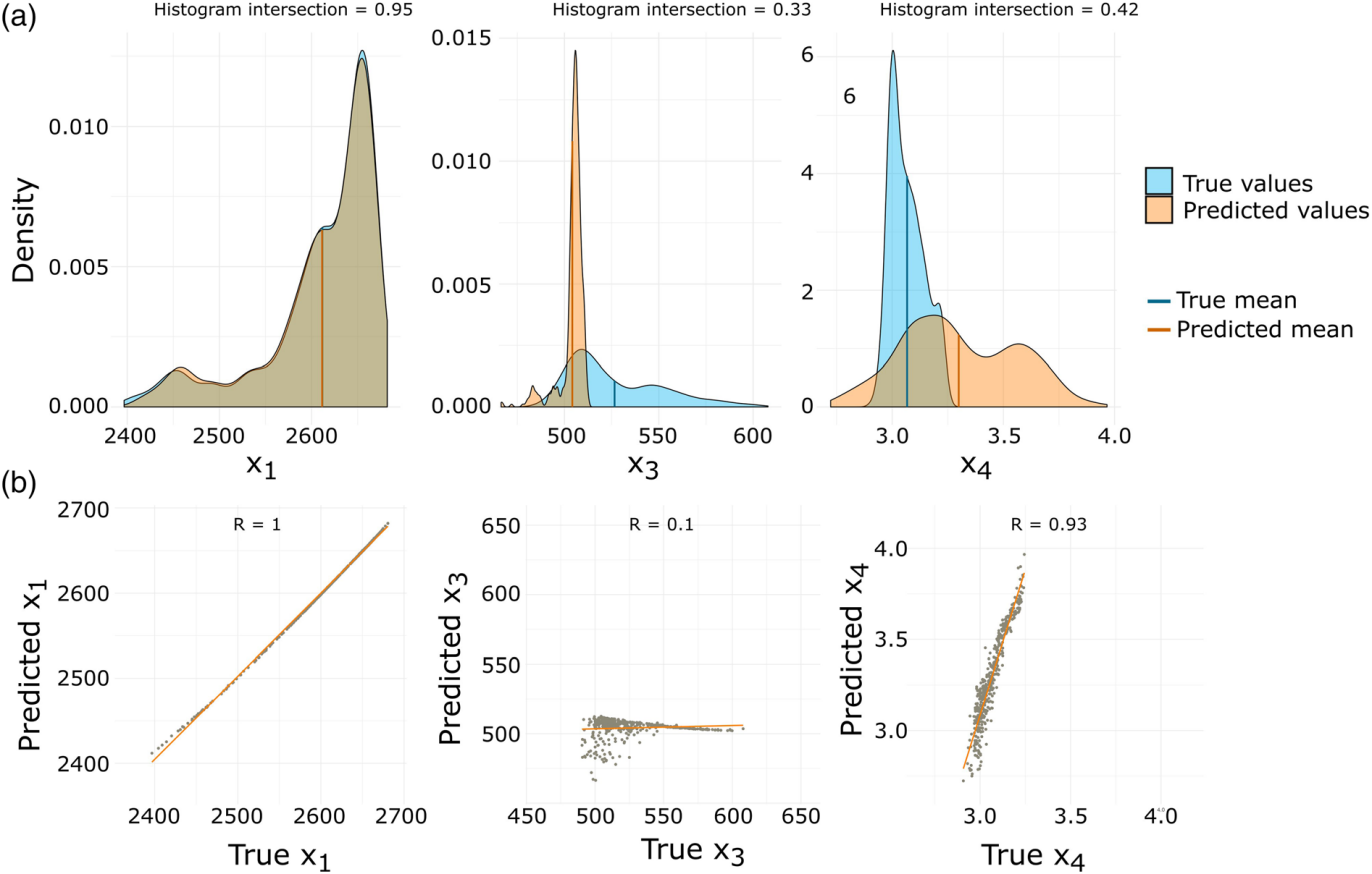
Results from multicriteria FSO, using NSE for Q and S, which is controlled by the parameter X1. All three optimized d‐GR4J parameters are compared to the true parameters on the 2 km model scale. (a) Estimated and true parameter densities with their mean values and corresponding histogram intersection. (b) Scatterplots of true versus estimated parameters and fitted linear model with Pearson correlation coefficients.

Figure 12 shows the estimated and true parameter distributions and scatterplots for all three multicriteria optimized parameters. The distribution of X1 is perfectly matched, and the predicted values have a nearly perfect linear relationship with a correlation coefficient of 1. However, X3 and X4 are less well matched compared to the single‐criteria FSO results with most X3 values being underestimated and most X4 values being overestimated. Due to the small, but existing variance in the estimation procedure, some runs performed better than the one shown here, but most results were similar to the one shown in Figure 12. We chose this run as it shows the general trend of estimation results.

A comparison of maps of the estimated parameter fields and the true parameter fields is shown in the supporting information (Figure S4).

### 4.5 Test 3: Multicriteria FSO Using NSE for Q and SPAEF for S

**Figure 13:**
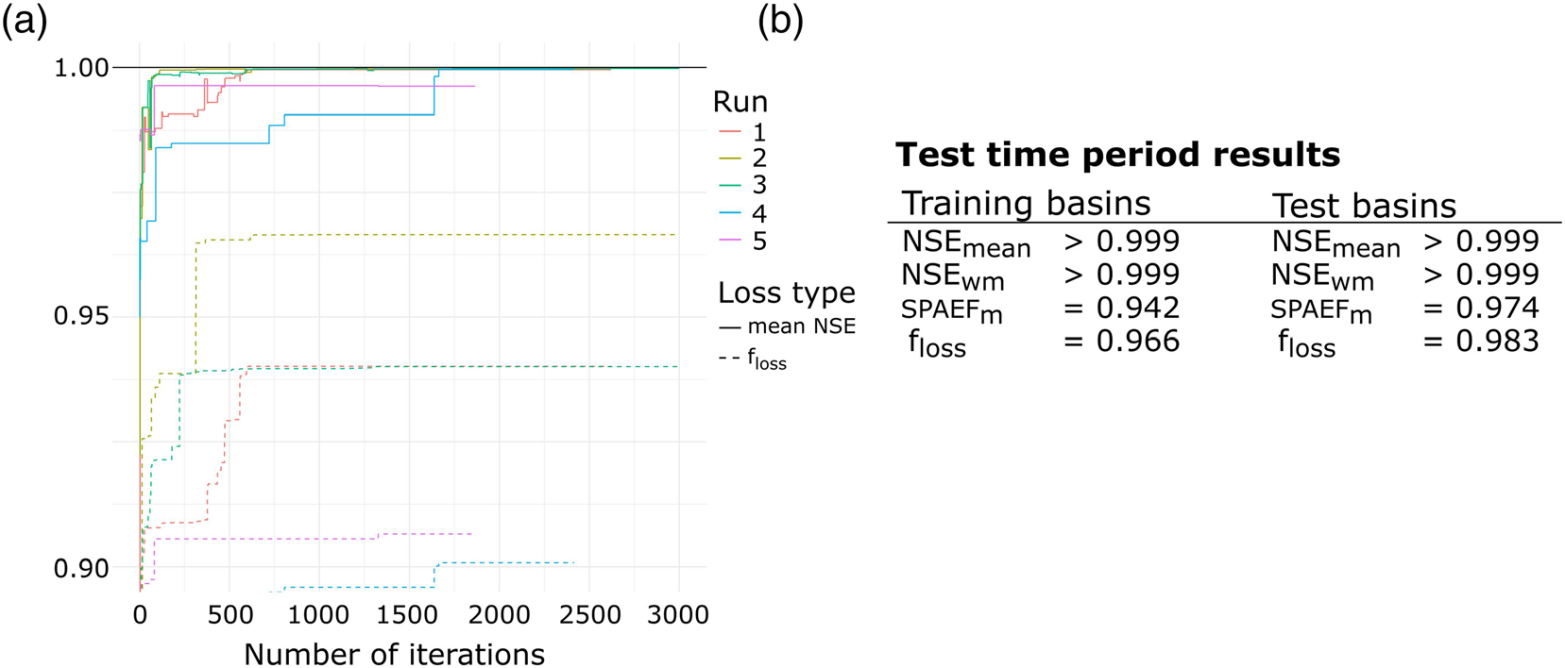
(a) Multicriteria FSO using NSE for Q and SPAEF for model state S training results for all five runs. (b) Summary of performance during the test time period for training and test basins.

Figure 13a shows the training performance of all five multicriteria FSO optimization runs using SPAEF for the d‐GR4J state S as additional optimization criteria. Therefore, the multivariate objective function consists of the NSE evaluated against Q and the SPAEF for the system state S. Similar to Test 2, we expect an improvement in estimating *f*_*tf*_ for X1. Compared to the previous tests, using SPAEF increased the variance in *f*_*loss*_. It is still very stable in regard to the resulting mean NSE.

For brevity, we show the detailed results of only one run. Due to the increased variance of the results, we chose the best training run (Run 2). Figure 13b shows the model performance in the test time period. It is notable that there is an increase in performance in the test basins and hence no overfitting to the training basins.

The comparison between true *f*_*tf*_ and multicriteria FSO estimated *f*_*tf*_ can be seen in Table 3. FSO was able to predict both of the spatial predictors of X1 correctly. The estimated transfer functions for the other two parameters however use different variables than the true functions.

**Table 3:** Comparison of the True *f*_*tf*_ and the Multicriteria FSO Using SPAEF Estimated *f*_*tf*_

Parameter	True *f*_*tf*_	FSO estimated *f*_*tf*_
X1	0.5+**evi** · 1.5+exp(**bdim**) · 0.9	0.7+exp(**evi**)+exp(**bdim**)
X3	−1.3 − log(**slope**)	0.7+*evi*/log(*elevation*)
X4	−1.5 − log(hand) · 0.2 − **slope** · 1.5	1.5/log(*elevation*)

**Figure 14:**
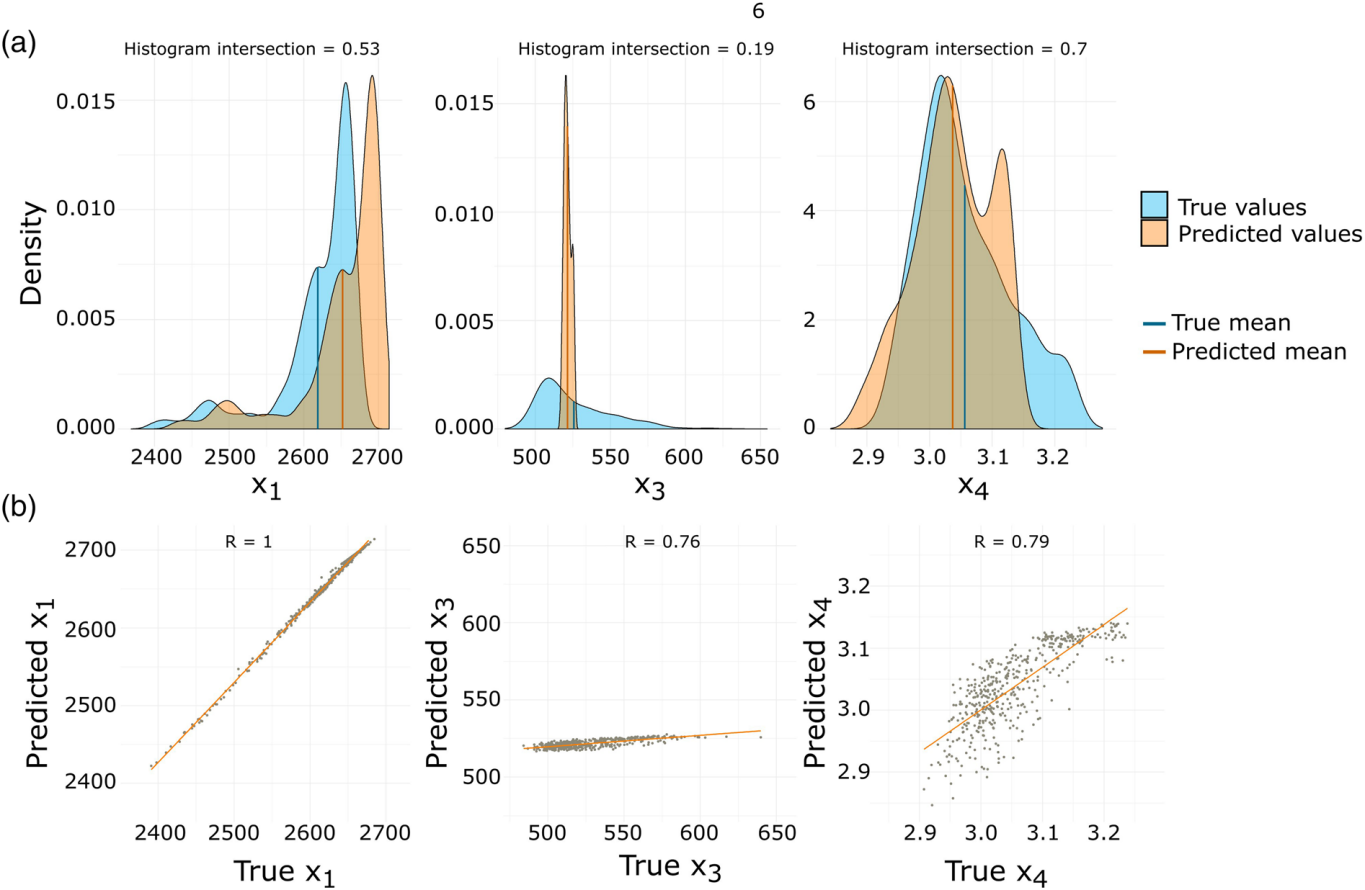
Results from multicriteria FSO using NSE for Q and SPAEF for model state S. All three optimized d‐GR4J parameters are compared to the true parameters on the 2 km model scale. (a) Estimated and true parameter densities with their mean values and corresponding histogram intersection. (b) Scatterplots of true versus estimated parameters and fitted linear model with Pearson correlation coefficients.

Figure 14 shows the estimated and true parameter distributions and scatterplots for all three SPAEF multicriteria optimized parameters. The values of X1 are perfectly matched with a correlation coefficient of 1, even though the distribution shows a slight shift to larger values. However, this shift (approximately 50 mm) accounts for only 1.25% of the overall range of possible X1 values ([1, 4,000]). X3 and X4 are better matched compared to the multicriteria FSO results using NSE. Both have approximately the same mean as the original distribution. The distribution of X4 is well matched, while it seems to be sufficient to get the right mean of X3 to have a nearly perfect performance.

## 5 Discussion and Conclusion

In this study, we present a method to automatically estimate parameter transfer functions for distributed hydrological models. Defining parameters as functions of the geophysical properties of a basin results in an increased physical interpretability of the model parameters, seamless parameter fields, and the possibility of prediction in ungauged basins. Our approach is based on the compression of functions from a CFG into a searchable continuous space (function space), which subsequently can be used for continuous optimization.

To demonstrate the predictive ability of FSO, we conducted a case study using synthetic data to avoid any influence of potential sources of errors, such as measurements errors and model assumptions, on the estimation procedure. The underlying true transfer functions were defined a priori and used for generating synthetic parameter fields that, in combination with the rainfall‐runoff model d‐GR4J, result in synthetic runoff and storage data.

We demonstrated that the developed function space has the desired properties of being “searchable” and that our chosen model parameters are sensitive. FSO is then tested in a case study using synthetic parameter fields and corresponding synthetic runoff and/or storage data.

The case study consists of two tests. First, we apply a single‐criteria calibration, optimizing the transfer functions only on runoff data in the calibration procedure. Second, this is then extended to additionally include spatially distributed time series of storage data in a multicriteria optimization. For both tests we could find transfer functions that produce a nearly perfect discharge prediction with an NSE of 0.999 in “ungauged” basins.

The results of the single‐criteria optimization show that FSO can find transfer functions that result in a perfect match for runoff and that FSO results are stable and multiple runs vary only insignificantly in their resulting mean basin NSE.

The results of both multicriteria optimization showed an increasing performance when estimating the parameter (X1), which is associated with the storage observations that are added to the loss function. Looking at the results of the different optimization runs, it is noteworthy that having the additional term in the loss function increases the difficulty of the optimization problem (see Figures 11a and 13a). We could observe only a slight improvement in performance when using NSE or SPAEF to evaluate the fit of the predicted model state. However, using SPAEF increased the parameter value fit of the two parameters not related to state S (X3 and X4). In a real‐world application it will most likely not be possible to use the NSE, since it assumes the same units of model states and spatial observations. Consequently, a multicriteria loss using the SPAEF will most likely be the applicable approach for most problems. Additionally, it should be noted that using both states S and R in the optimization further improved performance of FSO as shown in the supporting information (Figures S7 and S9).

Both single‐criteria and multiple‐criteria FSO did not show a decrease in performance in the test time period for the test basins. This shows that there is no overfitting on the training data and that prediction in ungauged basins is possible and performing as well as in a gauged basin. This is most likely due to the chosen penalty for complex functions in the loss function and the use of a weighted mean basin NSE value. Without the weighted NSE, single basins might have a bad fit while the overall training NSE is close to 1. Without the penalty for the length of the transfer functions, we could potentially find very complex functions that can approximate every other function without having any association to the process. The results let us conclude that, if FSO has a reasonable set of representative training basins, prediction in ungauged basins is possible.

Comparing the estimated transfer functions with the underlying true functions for the three chosen model parameters, it is notable that the most sensitive parameter X1 is usually estimated with the smallest deviation from the true parameter values.

From the results of the case study we can define the phenomena of transfer function equifinality, that is, nonunique best fitting transfer functions. We identified three main reasons for the occurrence of this form of equifinality. Reason one—parameter sensitivity: A low parameter sensitivity results in a reduced ability to identify the true transfer function, since small variations in parameter values are irrelevant for the resulting model loss. Reason two—information loss due to aggregation to the spatial scale of the model: Since aggregation generally results in loss of information, depending on the scale difference of observation and model scale, multiple transfer functions can potentially produce the same aggregated parameter field. Reason three—correlation of geophysical properties: Due to high correlation of geophysical properties (see section 3.1), different transfer functions using different spatial predictors can potentially produce similar parameter fields, for example, functions using sand instead of clay. Due to these three reasons, FSO estimated that transfer functions might differ in structure from the true underlying transfer functions but still perform well. Nevertheless, compared to the classical parameter equifinality, transfer function equifinality does not remove the physical interpretability of the estimated functions.

Assuming that a certain parameter can be described in mathematical form by some geophysical properties of a catchment, two important requirements are necessary for finding its true underlying transfer function. The CFG must be able to generate the function, and the correct geophysical parameters must be included in the CFG. In cases where these assumptions are violated, it can still be expected that FSO will find transfer functions which are associated with the physical processes described by the parameters. This is the case, because of the correlation of geophysical properties. Meaning, that even if we have not included the “correct” geophysical properties, we might still produce the correct parameter fields. Hence, we can assume association between model parameters and geophysical properties, even if there is not causality. This certainly increases the range of geophysical properties that can be used, but it is still necessary to have some that are related to the process described by a model parameter. Removing possibly unreleated geophysical properties or geophysical properties that are prone to measurement errors and spatial inconsistencies can also be an approach to increase physical realism. It would also be possible to add different weights to geophysical properties by adapting the function complexity penalty. This could be valuable for a real‐world application; however, in case of a synthetic case it might be assumed that geophysical properties with large measurement uncertainties are less likely to be chosen even without introducing additional weights.

Regarding the CFG, our results show that it is possible to include a wide range of different functions and function complexity in the CFG and still be able to search through the resulting function space. It is thus possible to define a (very) large space of possible functions for FSO and therefore have a high probability of including the true or a sufficiently approximating function in it.

Knowing the restrictions due to equifinality and assumptions related to FSO, we could show that the multicriteria tests increased its predictive capability for the state related parameter. FSO was able to estimate transfer functions which included the correct spatial predictors and had either the exact same or a very similar parameter field on the model scale compared to the true one. Therefore, similar to other studies that showed an increased model performance by using multicriteria parameter estimation, we could demonstrate an improvement in the search for a transfer function. The only disadvantage resulting from using multicriteria optimization is the increased complexity of the optimization task, which potentially increases the number of iterations needed.

In this study we could show that it is generally possible to estimate true underlying parameter fields with mathematical equations. However, it is important to keep in mind that the physical realism of the resulting functions does not solely rely on the here proposed method but also to a main part on the hydrological model definition and available data. This is especially relevant for large‐scale complex process‐based hydrological models, and it can be assumed that optimization restrains in the form of multicriteria optimization are necessary to find physical meaningfull relationships. Furthermore, FSO will only be able to find transferable functions when applied on a wide range of catchments. Consequently, the case where a given relationship is by chance only apparent in a certain catchment will not give too much weight to an unrelated function. It could still be the case that a given function is only applicable in a certain climatic region; however, this would still not result in a physical unrelated transfer function. We hope that this study can be a methodological starting point to further enhance data‐driven methods for process‐based hydrology.

## 6 Outlook

So far, the FSO performance has been purely evaluated in a synthetic setting, where all transfer functions, resulting parameter fields, as well as discharge and internal states are precisely known for any location and time step. Such a setting allows to test the principle functioning of the method and to explore potentials and limitations. In future work, the FSO method as presented here will be applied to more complex hydrological models using also real runoff data and spatial observations of relevant hydrological variables. This will provide further insight in the predictive capabilities of the FSO and the difficulties of estimating transfer functions in a real‐world setting. The application of the FSO to more physically based hydrological models will explore the potential of the FSO to identify and extract well‐known transfer function concepts as have been developed in the area of soil physics. The extension of the FSO application to larger regions will allow testing the transferability of derived model parameter transfer functions to “unkown” regions. The larger spectrum of geophysical properties will enable a more robust estimation of transfer functions and reduce the uncertainties in derived parameter fields.

However, an application of the FSO to larger regions with different climate conditions and land surface characteristics will potentially require more complex process descriptions, with more transfer functions to be estimated. In this context, it will be interesting to evaluate what type of additional spatially distributed data (e.g., time series of remote sensing data and derived products; see, e.g., Müller et al., 2014) might be able to improve the identification of transfer functions within the FSO framework and to reduce related uncertainties.

It would also be interesting to compare the performance of other symbolic regression methods (such as Genetic Programming) to benchmark our results.

## Supporting information

Supporting Information Figure S1

## Data Availability

The R code used to generate all results for this publication can be found in Feigl (2020). The data for geophysical properties are available from Feigl et al. (2020), and the discharge data used in this study are available online (https://www.ehyd.gv.at). The meteorological data from the INCA data set cannot be made public, because the rights belong to the Zentralanstalt für Meteorologie und Geodynamik (ZAMG). It can be acquired from https://www.zamg.ac.at website.
