# Supplementary material for: Function Space Optimization: A Symbolic Regression Method for Estimating Parameter Transfer Functions for Hydrological Models
Source: Water Resour Res. 2020 Oct 6;56(10):e2020WR027385. doi: 10.1029/2020WR027385 (PMC7583385; doi:10.1029/2020WR027385)
Supplement: Supplementary file 1 — Supporting Information Figure S1 [file WRCR-56-e2020WR027385-s001.pdf]

AGU PUBLICATIONS

Water resources Research

Supporting Information for

Function Space Optimization (FSO): A symbolic regression method for estimating parameter transfer functions for hydrological models

M. Feigl[1], M. Herrnegger[1], D. Klotz[1,2], K. Schulz[1]

[1]Institute for Hydrology and Water Management, University of Natural resources and Life Sciences, Vienna, Austria

[2]LIT AI Lab & Institute for Machine Learning, Johannes Kepler University Linz, Linz, Austria

Contents of this file

Text S1 to S2
Figures S1 to S9

Introduction

The following supporting material contains additional information and results that could not be covered in the main manuscript. Text S1 gives a short theoretical description of the variational autoencoder (VAE) architecture. Text S2 describes the FSO VAE in detail and explains how functions are sampled from Function Space. Figure S1 shows the FSO VAE architecture. Figure S2 shows the CFG that was used in the case study. Figures S3, S4 and S5 show the true and estimated parameter fields of FSO for single-criteria and multi-criteria optimization. Figures S6 and S7 show the FSO results of the NSE multi-criteria optimization using the time-series of state R and both S and R. Figures S8 and S9 show the FSO results of the SPAEF multi-criteria optimization using the time-series of state R and both S and R.

Text S1.

A variational autoencoder (VAE) is a neural network architecture consisting of two different neural networks, an encoder $p_{enc}(z|x)$ and a decoder $p_{dec}(x|z)$. Here p denotes a probability function. This notation explicitly refers to the stochastic nature of a VAE. The encoder transforms information of inputs x to a latent vector space z, while the decoder reconstructs x from z. VAEs assume a stochastic latent space, which is usually chosen to be gaussian:

$$z \sim \mathcal{N}(\mu|x, \Sigma|x),$$

with mean μ and a diagonal covariance matrix Σ.

For training a VAE, the Kullback-Leibler Convergence between the learned latent distribution $p_{enc}(z|x)$ and its desired distribution p(z) is used as penalty term in the loss function. Therefore, the optimization is forced to set the parameters of the network so that the values of z are distributed around the center and not located sparsely in the continuous space.

Text S2.

A detailed representation of the FSO VAE is shown in Figure S1. Both encoder and decoder consist of two parts, each for one of the two different inputs. The encoding of function strings is done by an embedding layer to create a matrix representation for every function, followed by a combination of multiple convolutional neural network (CNN) layers and two dense layers. The decoding of the function strings is done by a combination of 32 Dense layer, a Bi-directional Long short-term memory (LSTM) network and one final dense layer. This dense layer has a softmax activation function and thus returns a matrix of probabilities with the dimensions: (total number of unique symbols) x (max. number of symbols in function). Each column of this matrix sums up to 1 (property of softmax function) and each entry gives the probability of a certain symbol (row) to belong to a certain position (column) of the resulting string.

The encoding and decoding of the functions parameter distributions consists of 3 consecutive dense layer with a selu (scaled exponential linear unit) activation function. The decoding part has one additional dense layer with a linear activation function to create the output for the distribution values.

The middle part in Figure S1 shows the sampling and the resulting latent space. In the sampling part the information of the function string and the functions parameter distribution get concatenate and used as an input for two 6 dimensional dense layers with linear activation functions. Those two layers output the mean and standard deviation for the following sampling layer. The latent space, denoted as vector z in Figure S1, is thus created by sampling from a multivariate normal distribution using the mean and standard deviation (assuming a diagonal covariance matrix) from the two previous layers. The following decoding layers have only z as input for reconstructing both inputs.

The loss function used for the FSO VAE consists of a reconstruction loss (for the function strings and the parameter distribution) and the Kullback-Leibler (KL) divergence (see Text S2). The reconstruction loss is the sum of the cross entropy of the function string reconstruction and the mean squared error (MSE) of the parameter distribution reconstruction. MSE and the KL divergence have an additional weighting factor to balance their importance during training. With that we can define our loss function as

$$\boldsymbol{Loss} = \boldsymbol{E}[\boldsymbol{log}\,\mathbf{p_{dec}}(\boldsymbol{x}|\boldsymbol{z})] - \boldsymbol{\beta} \times \boldsymbol{D_{KL}}[\mathbf{p_{enc}}(\boldsymbol{z}|\mathbf{x})||\mathbf{p}(\boldsymbol{z})] + \boldsymbol{\alpha} \times \boldsymbol{E}[(\boldsymbol{d} - \widetilde{\boldsymbol{d}})^{\mathbf{2}}]. \quad (1)$$

The variable x denotes the function string inputs, z denotes the latent space representation of the inputs, d denotes the vector of quantiles representing the parameter distribution inputs and $\tilde{d}$ its reconstructed version. $\mathrm{p_{dec}}$ and $\mathrm{p_{enc}}$ denote the probability function resulting from the encoder and decoder network, respectively. The first part of the loss function, $E[log\,\mathrm{p_{dec}}(x|z)]$, denotes the expectation of the cross-entropy loss. The second part, $\beta \times D_{KL}[\mathrm{p_{enc}}(z|\mathrm{x})||\mathrm{p}(z)]$ is the Kullback-Leibler divergence between the distribution resulting from the encoder $\mathrm{p_{enc}}(z|\mathrm{x})$ and the assumed distribution of the latent variable $\mathrm{p}(z)$ multiplied by a factor β. The third part is the MSE loss multiplied by a factor α. Both factors α and β were chosen after some trial and error to be 100 and 1000, respectively. The large factor for α was necessary since the FSO VAE was trained on standardized quantiles which resulted in the MSE being diminishing small compared to the other losses.

After training the VAE with a sufficiently large sample from the CFG, only the function string decoder part of the VAE is used to generate transfer functions from the latent space. This is done by taking the symbols with maximum probability from the softmax output of the function string decoder.

Even with the variational constraint of the VAE architecture, occasionally invalid functions are generated. This is due to the sensitive structure of mathematical functions, where missing parentheses or missing operation symbol between other symbols result in an invalid function. To cope with this, in any point of the function space where the maximum probabilities do not produce a valid function, we apply a robust estimation of the most probable valid transfer function associated with this point. For this we draw 2000 samples using the softmax probabilities for each symbol and choose the most often occurring valid transfer function.

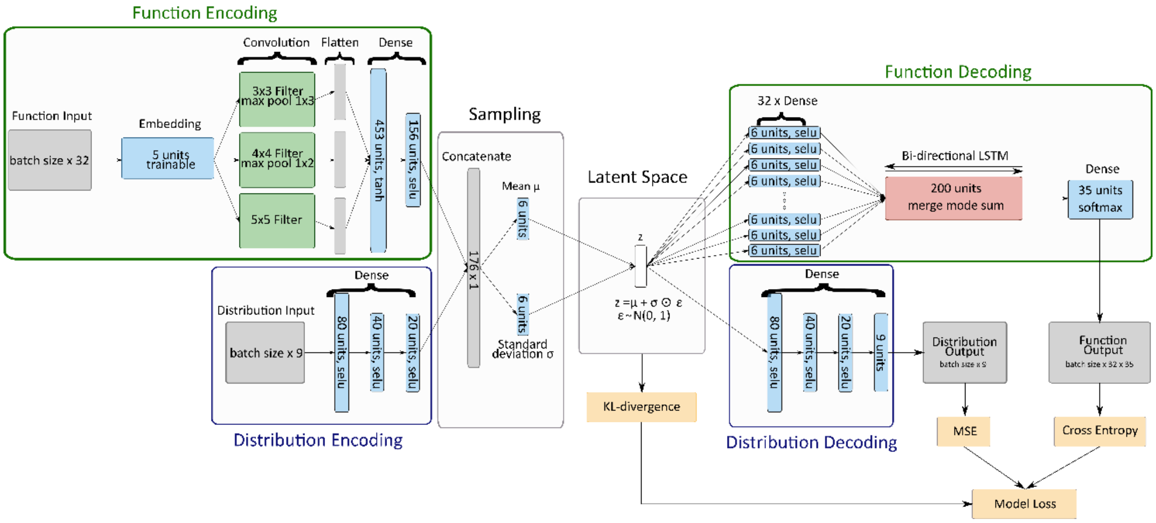

Figure S1. Detailed structure of the FSO variational autoencoder and corresponding losses. Dimensions are given for an example of a set of training strings with a maximal length of 32 symbols.

tf = **numeric** * **eq** + **numeric** | **eq** + **numeric** | **eq**
eq = **fs** | **eq op fs** | **eq op numeric** | **fs op numeric**
fs = **sp** | **f**(**sp**) | **sp op sp** | **numeric**
f = exp | log
op = + | - | * | /
sp = slope | evi | sand | clay | elevation | hand | noise | bdim
numeric = [-1.5, 1.5]

Figure S2. The CFG used in the case study. It contains recursive arguments that could loop until a maximum recursive depth of 3. The numeric values are given as a sequence of discrete values in the interval [-1.5, 1.5] with a step size of 0.1.

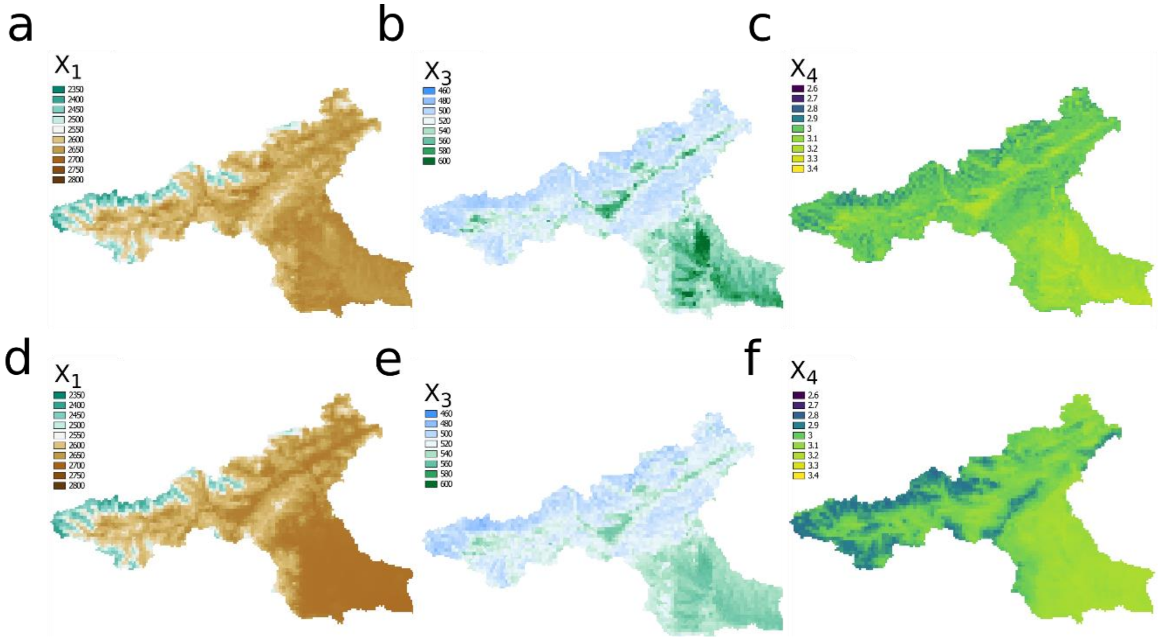

Figure S3. Comparison of parameter fields created by true (**a**, **b**, **c**) and FSO (**d**, **e**, **f**) estimated transfer functions for single-criteria FSO.

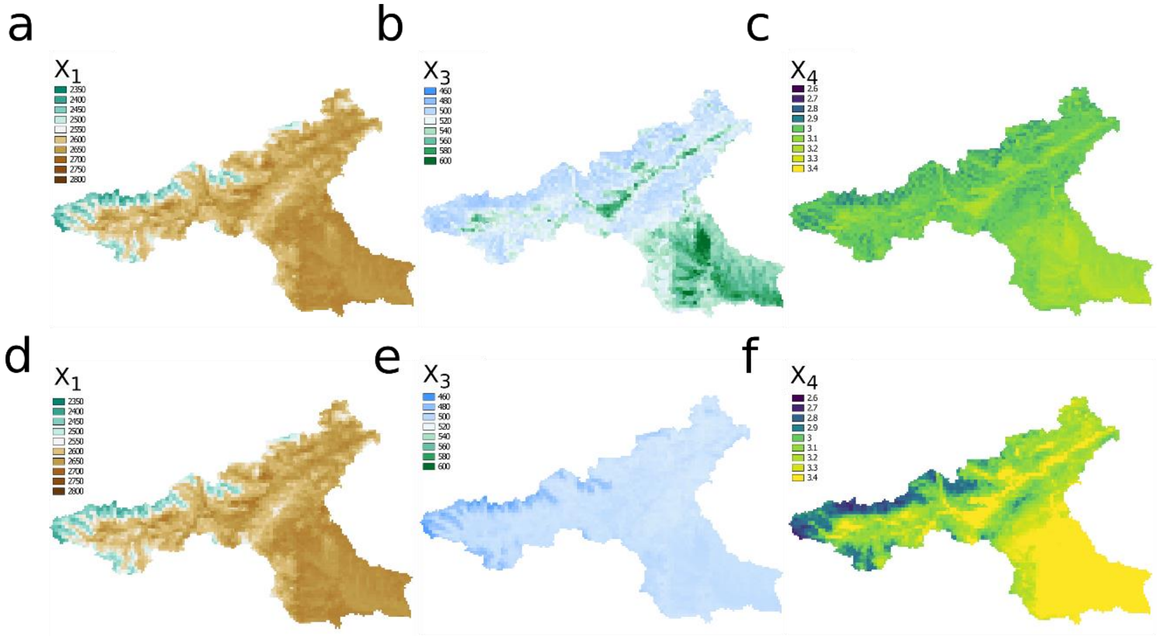

Figure S4. Comparison of parameter fields created by true (a, b, c) and FSO (d, e, f) estimated transfer functions for multi-criteria FSO using NSE and the time-series of state S.

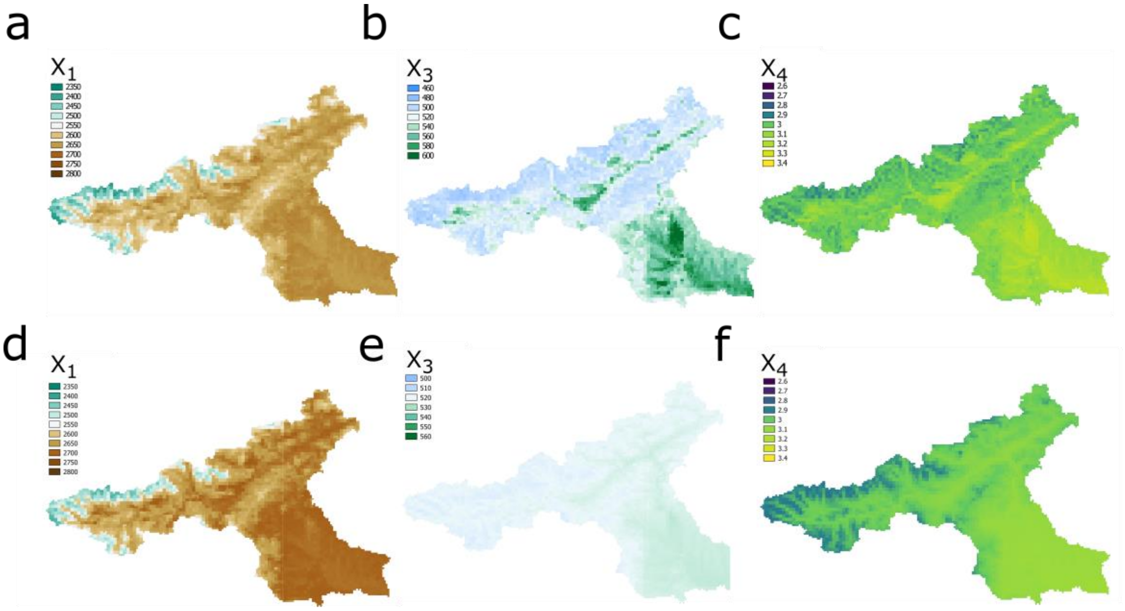

Figure S5. Comparison of parameter fields created by true (a, b, c) and FSO (d, e, f) estimated transfer functions for multi-criteria FSO using SPAEF and the time-series of state S.

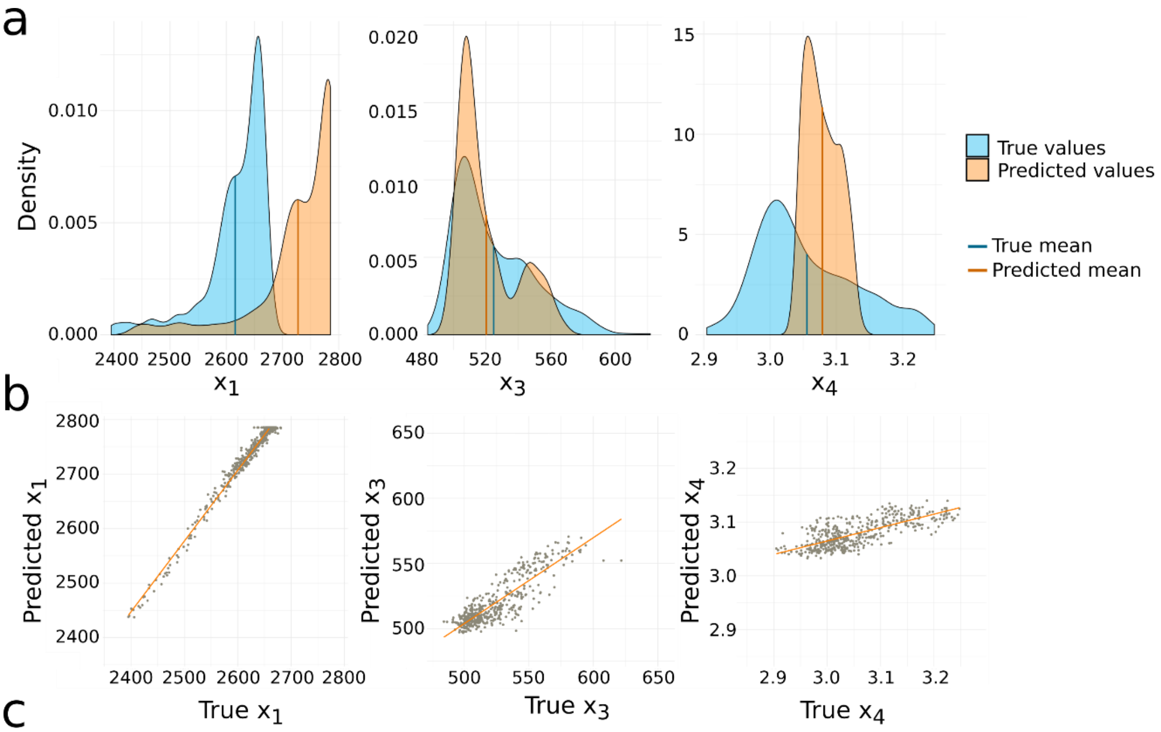

c

	X1	X3	X4	Iterations	Train NSE	Test NSE
True functions	$0.5 + \text{evi} \times 1.5 + \exp(\text{bdim}) \times 0.9$	$-1.3 - \log(\text{slope})$	$-\log(\text{hand}) \times 0.2 - \text{slope} \times 1.5 - 1.5$			
FSO	$1.1 \times \text{bdim} + 3$	$\text{slope} - (0.7 \times \exp(\text{slope}) + \log(\text{slope}) \div 1.5)$	$\exp(\text{hand}) - 1$	1484	0.999	1

Figure S6. NSE multi-criteria optimization results using the time-series of state R for all 3 optimized d-GR4J parameters on the 2 km model scale. **a** Estimated and true parameter densities with their mean values. **b** Scatterplots of true vs. estimated parameters and fitted linear model. **c** Comparison of true and estimated transfer functions with numbers of iterations and training and test NSE values.

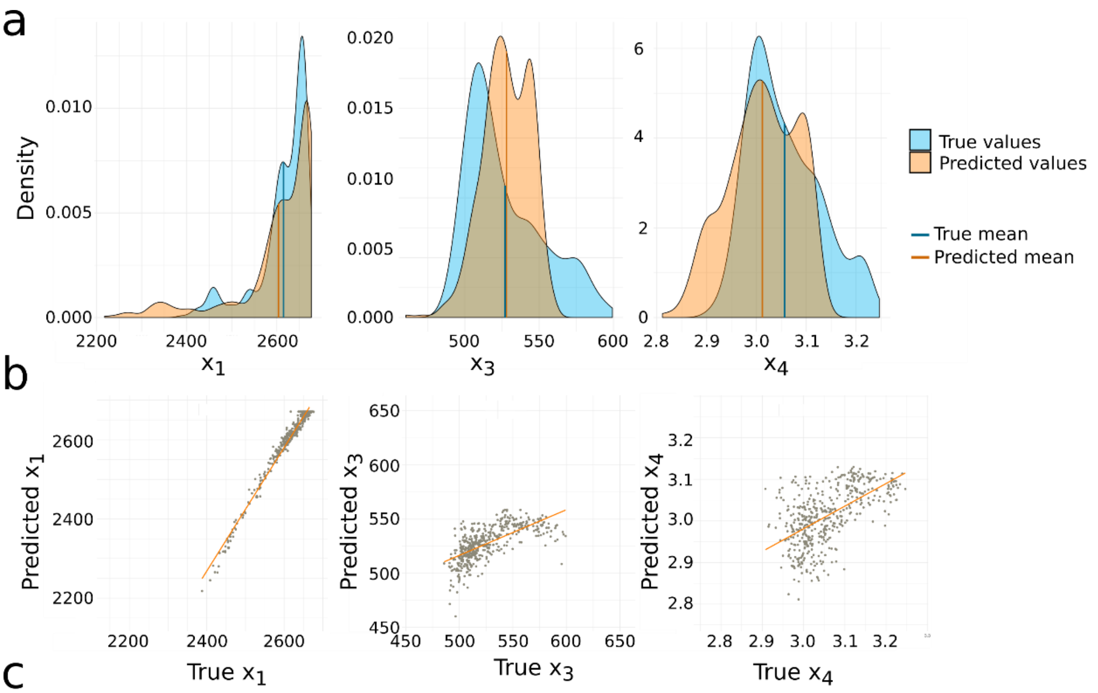

c

	X1	X3	X4	Iterations	Train NSE	Test NSE
True functions	0.5 + evi × 1.5 + exp(bdim) × 0.9	− 1.3 − log(slope)	− log(hand) × 0.2 − slope × 1.5 − 1.5			
FSO	0.7 + 1.1 × exp(bdim) + log(bdim)	exp(clay) + noise ÷ sand − 0.6 ÷ evi	evi + log(clay) − log(bdim)	2076	1	1

Figure S7. NSE multi-criteria optimization results using the time-series of state R and S for all 3 optimized d-GR4J parameters on the 2 km model scale. **a** Estimated and true parameter densities with their mean values. **b** Scatterplots of true vs. estimated parameters and fitted linear model. **c** Comparison of true and estimated transfer functions with numbers of iterations and training and test NSE values.

a

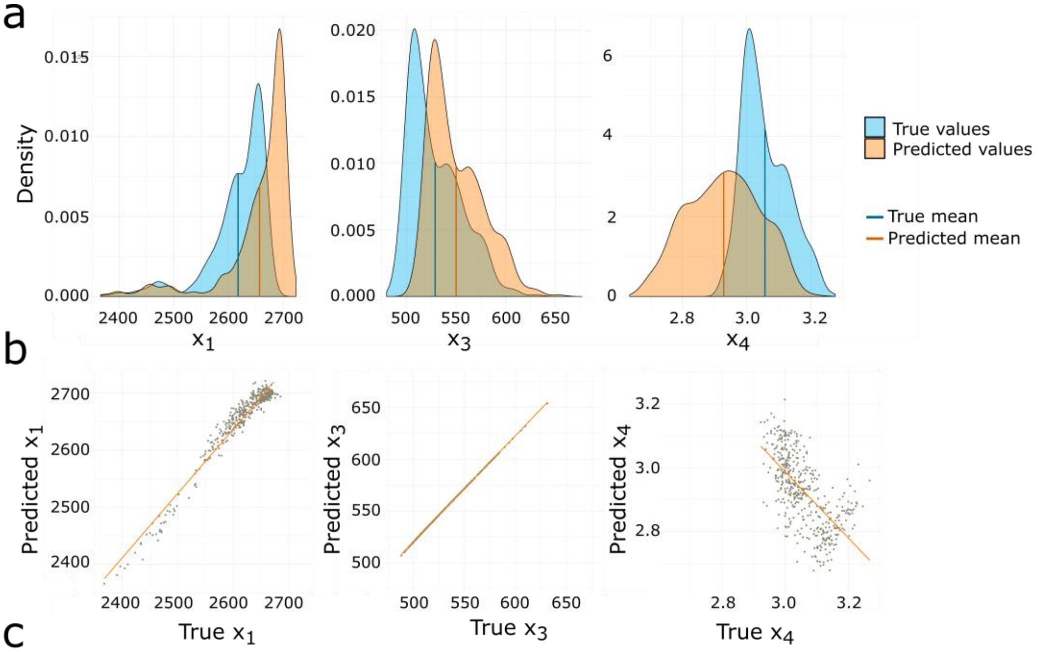

c

	X1	X3	X4	Iterations	Train NSE	Test NSE
True functions	$0.5 + evi \times 1.5 + \exp(bdim) \times 0.9$	$-1.3 - \log(slope)$	$-\log(hand) \times 0.2 - slope \times 1.5 - 1.5$			
FSO	$bdim + \exp(bdim) + slope$	$slope \times (0.8333333 - \exp(hand)) - (0.8 + \log(slope))$	$0.7 + \exp(clay)^2 \div \log(evi)$	2266	0.998	0.999

Figure S8. SPAEF multi-criteria optimization results using the time-series of state R for all 3 optimized d-GR4J parameters on the 2 km model scale. **a** Estimated and true parameter densities with their mean values. **b** Scatterplots of true vs. estimated parameters and fitted linear model. **c** Comparison of true and estimated transfer functions with numbers of iterations and training and test NSE values.

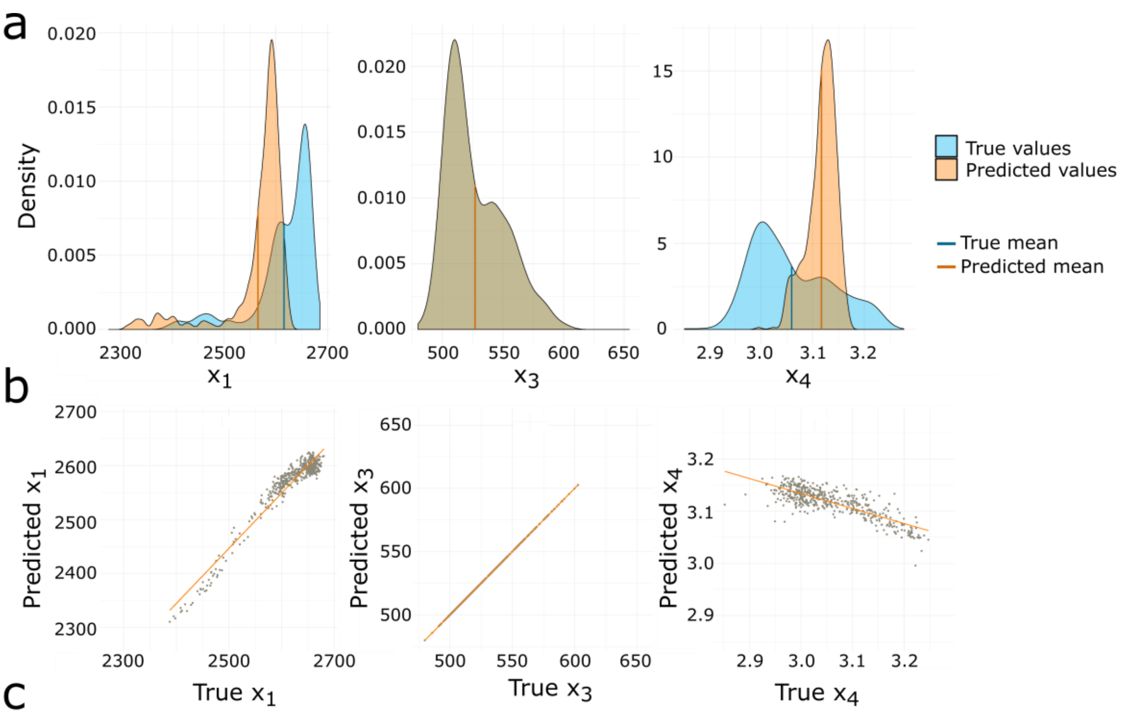

c

	X1	X3	X4	Iterations	Train NSE	Test NSE
True functions	0.5 + evi × 1.5 + exp(bdim) × 0.9	− 1.3 − log(slope)	− log(hand) × 0.2 − slope × 1.5 − 1.5			
FSO	bdim × sand + exp(bdim)	− 1.3 − log(slope)	log(evi) ÷ exp(elevation) + slope	1585	0.999	1

Figure S9. SPAEF multi-criteria optimization results using the time-series of state R and S for all 3 optimized d-GR4J parameters on the 2 km model scale. **a** Estimated and true parameter densities with their mean values. **b** Scatterplots of true vs. estimated parameters and fitted linear model. **c** Comparison of true and estimated transfer functions with numbers of iterations and training and test NSE values.
